# Soybean-Derived Soluble Fiber Enhances Antimicrobial Proteins in the Mouse Small Intestine via a Tuft Cell–Group 2 Innate Lymphoid Cell Axis

**DOI:** 10.1021/acs.jafc.6c01854

**Published:** 2026-06-03

**Authors:** Arslan Ahmad, Bambang Dwi Wijatniko, Cláudia Domingos Mapure Siueia, Chisato Yanagi, Yoshiki Ishii, Shodai Ishikawa, Ryo Inoue, Dina Mustika Rini, Noriyuki Yanaka, Takuya Suzuki

**Affiliations:** 1 Graduate School of Integrated Sciences for Life, 12803 Hiroshima University, 1-4-4 Kagamiyama, Higashi-Hiroshima 739-8528, Japan; 2 Department of Food and Agricultural Product Technology, Universitas Gadjah Mada, Yogyakarta 55281, Indonesia; 3 Faculty of Agriculture, Setsunan University, 45-1 Nagaotoge-cho, Hirakata 573-0101, Japan

**Keywords:** dietary fiber, antimicrobial proteins, tuft cells, type 2 immunity

## Abstract

Water-soluble soybean fiber (WSSF) exerts health benefits beyond its fermentability, but its direct effects on epithelial–immune interactions remain unclear. We investigated whether WSSF regulates antimicrobial proteins (AMPs) in the small intestine and the underlying mechanisms. C57BL/6J and tuft cell–deficient Pou2f3-knockout mice were fed control or WSSF-supplemented diets, and gene and protein expression and cecal microbiota were analyzed. WSSF upregulated the epithelial AMPs SPRR2A, RELMβ, and ANG4 in the jejunum and ileum. Pharmacological inhibition of group 2 innate lymphoid cells (ILC2) or TRPM5 attenuated AMP induction and reduced IL-25, IL-13, tuft cell markers, and STAT6 phosphorylation. WSSF also failed to induce AMPs or type 2 immune markers in *Pou2f3*-knockout mice, indicating a requirement for tuft cells. Finally, WSSF altered cecal microbiota composition in a tuft cell–dependent manner. These findings identify WSSF as a soybean-derived fiber that strengthens epithelial innate defense via a TRPM5–tuft cell–ILC2 axis.

## Graphical abstract

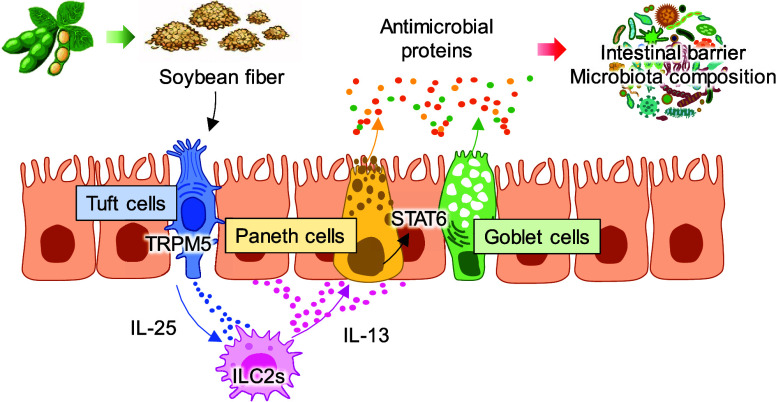

## 1 Introduction

Dietary fibers are widely recognized as essential components of a healthy diet and contribute to numerous physiological benefits, including glycemic control, lipid metabolism, and modulation of the gut microbiota.^1^ A large body of research has established that many of the health effects of dietary fibers are mediated through microbial fermentation and the production of short-chain fatty acids and other metabolites.^2^ However, emerging evidence suggests that certain dietary fibers can also exert microbiota-independent effects, directly influencing epithelial or immune cell functions. Despite this intriguing possibility, mechanistic studies examining the direct cellular actions of dietary fibers remain extremely limited, and the underlying pathways are poorly understood.

The intestinal barrier plays a critical role in maintaining host–microbe homeostasis and preventing the translocation of pathogens and harmful luminal substances.^3,4^ Antimicrobial proteins (AMPs), produced by various epithelial lineages including Paneth and goblet cells, are key effector molecules that reinforce barrier integrity by inhibiting pathogenic bacteria and shaping microbial composition.^5^ Among AMPs, ANG4 is mainly produced by goblet cells, while resistin-like β (RELMβ) and small proline rich protein 2a (SPRR2A) are produced both by Paneth and goblet cells. Impaired AMP expression has been associated with inflammatory bowel disease, food allergies, metabolic syndrome, and susceptibility to infection.^6^ Therefore, identifying food-derived components that enhance AMP production represents a promising strategy for supporting intestinal health and preventing disease.

Recent advances in mucosal immunology have highlighted tuft cells as critical epithelial sentinel cells that monitor the intestinal lumen. Tuft cells detect parasites and parasite-derived metabolites, as well as various luminal chemical cues, and respond by secreting interleukin-25 (IL-25). IL-25 activates group 2 innate lymphoid cells (ILC 2s), which subsequently produce IL-13.^7^ IL-13 drives epithelial remodeling, including goblet and tuft cells expansion, and promotes the expression of AMPs through STAT6-dependent signaling pathways.^8,9^ This tuft cell–ILC2–IL-13 axis represents a key epithelial–immune circuit linking luminal sensing to innate defense.^9^ While several microbial metabolites have been identified as activators of tuft cells, interactions between dietary components—particularly dietary fibers—and tuft cells remain largely unexplored.

Water-soluble soybean fiber (WSSF), prepared from defatted soybeans, is widely incorporated into food products owing to its favorable fermentability, texture-modifying properties, and potential health benefits.^10^ WSSF is a soybean-derived soluble polysaccharide composed mainly of homogalacturonan and rhamnogalacturonan domains, with galactan and arabinan side chains. It has an average molecular weight of approximately 500,000 and exhibits low viscosity. Dietary supplementation with WSSF has been reported to exert several physiological effects, including modulation of lipid metabolism, mineral absorption, and immune responses.^10–12^ In other soluble dietary fibers and plant-derived polysaccharides, structural properties such as molecular weight, branching pattern, and side-chain composition have been suggested to influence their biological and immunomodulatory activities. Although such structure–activity relationships have not been fully established for WSSF, these findings raise the possibility that the characteristic polysaccharide structure of WSSF may contribute to its physiological effects. A previous study also showed that WSSF ameliorated experimentally induced colonic inflammation by suppressing the TLR4/NF-κB signaling pathway.^13^ However, despite its increasing use as a functional food ingredient, little is known about how WSSF influences epithelial–immune crosstalk in the small intestine, particularly through microbiota-independent mechanisms.

Therefore, the objective of this study was to determine whether WSSF regulates antimicrobial protein expression in the small intestine and to elucidate the immunological pathways involved. Elucidating these mechanisms will provide new insight into how soybean-derived dietary fiber contributes to intestinal epithelial defense and may support the development of functional foods that enhance gut health through epithelial–immune modulation.

## 2 Materials and Methods

### 2.1 Chemicals

Water-soluble soybean fiber (WSSF; SOYAFIBE-SDA100) was kindly provided by Fuji Foundation for Protein Research (Osaka, Japan). Unless otherwise specified, all other chemicals were obtained from Nacalai Tesque (Kyoto, Japan) or FUJIFILM Wako Pure Chemical Corp. (Osaka, Japan).

### 2.2 Mice

All animal procedures were approved by the Animal Care Committee of Hiroshima University (approval no. C24–28) and complied with Hiroshima University’s Guidelines for the Care and Use of Laboratory Animals, the National Research Council’s Guide for the Care and Use of Laboratory Animals, and the ARRIVE guidelines. Seven-week-old female C57BL/6J mice were purchased from Jackson Laboratory Japan (Yokohama, Japan). *Pou2f3* knockout (*Pou2f3*-KO) mice (B6.129-Pou2f3^tm1Abek^; RBRC05254) were obtained from the RIKEN BRC through the National BioResource Project (MEXT/AMED, Japan). Homozygous KO mice and wild-type littermates were generated by breeding heterozygous mice. Mouse genomic DNA was prepared from tail biopsies by an alkaline extraction method. Genotyping was carried out by PCR using KOD One PCR Master Mix (TOYOBO, Osaka, Japan) in accordance with the manufacturer’s protocol. Primer sequences for identification of the wild-type and knockout alleles are provided in Table S1. Representative images of the genotyping PCR products separated by gel electrophoresis are presented in Supplemental Figure S1. Mice were housed under standard conditions (22 ± 2 °C; 40–60% humidity; 12-h light/dark cycle, lights on 08:00–20:00) with free access to tap water and a fiber-free AIN-93G diet (Table S2) for at least 1 week before experiments. At the end of each study, mice were euthanized by exsanguination under isoflurane anesthesia.

### 2.3 Experimental Design

Four independent experiments (Experiments 1–4) were performed to evaluate the regulatory role of WSSF on intestinal AMP expression in mice. Female C57BL/6J mice were used in Experiments 1–3 to reduce variability in the initial evaluation of WSSF-induced epithelial–immune responses. In Experiment 4, male *Pou2f3*-KO mice and their wild-type littermates were used to clarify the contribution of tuft cells. Because male and female mice were not compared under the same experimental conditions, sex-specific effects of WSSF were not directly examined in this study.

#### 2.3.1 Experiment 1: Whole-Transcriptome and Molecular Analyses

To examine the impact of WSSF on small-intestinal gene expression, female mice were randomly assigned to three groups (control, 5% WSSF, and 10% WSSF; n = 6/group) and fed the respective diets for 5 days (Figure 1A). WSSF was incorporated by replacing an equal amount of starch (Table S2). A 5-day feeding period was selected as a short-term intervention to evaluate whether WSSF rapidly affects small-intestinal antimicrobial protein expression and related immune signaling pathways. Jejunal segments (1 cm distal to the ligament of Treitz) were collected for RNA-seq, quantitative reverse transcription-polymerase chain reaction (qRT-PCR), immunoblotting, and immunofluorescence. Ileal segments (2 cm proximal to the ileocecal junction) were analyzed by qRT-PCR and immunoblotting. Colonic segments (1 cm distal to the cecocolic junction) were used for qRT-PCR. Fresh fecal samples were collected on day 5 for short-chain fatty acid (SCFA) analysis.

**Figure 1:**
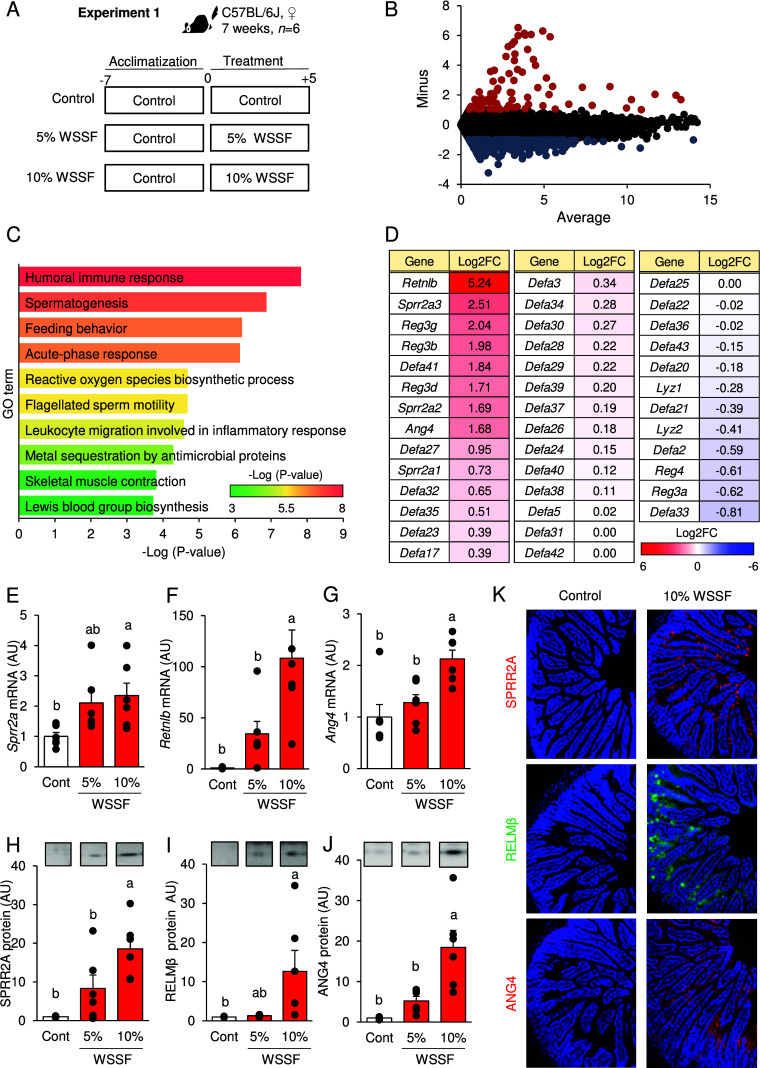
Soybean fiber enhances antimicrobial protein expression in the mouse small intestine. Data shown are from Experiment 1. Mice were fed control diets or diets supplemented with 5% or 10% water-soluble soybean fiber (WSSF) for 5 days, as illustrated in the experimental design (A). Jejunal tissues were analyzed by RNA sequencing (RNA-seq; B–D), quantitative reverse transcription-PCR (qRT-PCR; E–G), immunoblotting (H–J), and immunofluorescence analyses (K). An MA plot is shown using log_2_-transformed transcript-per-million (TPM) values (B). “Average” represents the mean expression levels of the control and 10% WSSF groups, whereas “minus” indicates the differences between these two groups. Each dot represents an individual gene; red dots indicate genes that were upregulated (>2-fold) or downregulated (<0.5-fold) in the 10% WSSF group compared with the control group. Gene Ontology (GO) enrichment analysis of the upregulated genes was performed using Metascape, and the top 10 GO terms ranked by *P* value are shown (C). Fold changes in antimicrobial protein gene expression in the 10% WSSF group relative to the control group are shown (D). mRNA expression levels of *Sprr2a*, *Retnlb*, and *Ang4* were quantified by quantitative reverse transcription-PCR (qRT-PCR; E–G), and protein expression levels were evaluated by immunoblotting (H–J). Representative immunofluorescence images of SPRR2A, RELMβ, and ANG4 in the jejunum are shown (K). Data are presented as mean ± s.e.m. Statistical significance was assessed using the Tukey–Kramer post hoc test or the Steel–Dwass test. Groups not sharing a common letter are significantly different (*p* < 0.05). AU: arbitrary unit.

#### 2.3.2 Experiment 2: ILC2 Inhibition

To determine the involvement of group 2 innate lymphoid cell (ILC2), the ILC2 inhibitor disulfiram (DSF) was administered.^14^ Female C57BL/6J mice were randomly divided into four groups: control, 10% WSSF, 10% WSSF + low dose DSF, and 10% WSSF + high dose DSF (Figure 4A). Mice in the three WSSF groups received a 10% WSSF diet for 5 days; the control group received the control diet (Table S2). Disulfiram was administered orally at 1.2 or 2.4 mg/kg body weight/day, starting 2 days prior to WSSF feeding (7 days in total). Control and WSSF groups received ultrapure water. Disulfiram doses were based on our previous study.^15^ Jejunal tissues were collected for qRT-PCR, immunoblot, and immunofluorescence.

#### 2.3.3 Experiment 3: TRPM5 Inhibition

To investigate the role of TRPM5 channels, which mediate IL-25 production in tuft cells, the TRPM5 inhibitor triphenylphosphine oxide (TPPO) was used.^16^ Female C57BL/6J mice were allocated into three groups: control, 10% WSSF, and 10% WSSF + TPPO (Figure 6A). Mice were fed the corresponding diets for 5 days (Table S2); TPPO was added at 1% (w/w) beginning 2 days before WSSF feeding. Jejunal segments were subjected to qRT-PCR, immunoblotting, and immunofluorescence.

#### 2.3.4 Experiment 4: Tuft Cell-Dependent Responses (*Pou2f3*-KO Model)

To clarify the contribution of tuft cells, male *Pou2f3*-KO mice and wild-type littermates were assigned to two diet groups each (control or 10% WSSF) and fed for 5 days (Figure 8A; Table S2). Jejunal and ileal tissues were collected for qRT-PCR, immunoblotting, and immunofluorescence. Cecal contents were analyzed for microbiota composition by 16S rRNA gene sequencing.

### 2.4 Whole Transcriptome Analysis of Mouse Jejunum

Jejunal segments from the control and 10% WSSF groups were processed for RNA-seq analysis. Total RNA was isolated from jejunal tissues using NucleoSpin RNA (Macherey-Nagel, Düren, Germany) according to the manufacturer’s instructions. Equal amounts of RNA from six samples in each group was pooled and processed as described previously.^15^ Mapped read counts were normalized to transcripts per million (TPM). RNA from six animals in each group was pooled and subjected to RNA-seq to obtain a representative transcriptomic profile for each group and to reduce the influence of interindividual variability at the screening stage. Because the pooled RNA-seq data set did not allow biological replicate-based statistical inference, RNA-seq was used primarily as an exploratory transcriptomic analysis. Key genes identified as differentially expressed by RNA-seq were subsequently validated by qRT-PCR using RNA from individual animals.

### 2.5 qRT-PCR Analysis

Total RNA was isolated from intestinal tissues using either NucleoSpin RNA (Macherey-Nagel) or Sepasol-RNA I Super G (Nacalai Tesque). cDNA was synthesized using the ReverTra Ace qPCR RT kit (TOYOBO). qRT-PCR was performed with the 2× Brilliant III Ultra-Fast SYBR Green QPCR Master Mix (Agilent Technologies) on a StepOne Real-Time PCR System (Thermo Fisher Scientific). Primer sequences are listed in Table S3. Relative expression levels were calculated using the 2^–ΔΔCt^ method with ribosomal protein S28 (*Rps28*) as the reference gene.

### 2.6 Immunoblot Analysis

Jejunal and ileal tissues (∼20 mg) were homogenized in 500 μL of lysis buffer [1% sodium dodecyl sulfate, 1% Triton X-100, 1% sodium deoxycholate, 30 mM Tris-HCl (pH 7.4), protease and phosphatase inhibitors] using a Polytron homogenizer (KINEMATICA, Luzern, Switzerland). Protein concentrations were determined using a BCA protein assay kit (FUJIFILM Wako Pure Chemical). Samples were mixed with 3× Laemmli buffer, heated at 95 °C for 10 min, separated by sodium dodecyl sulfate–polyacrylamide gel electrophoresis (20 μg protein/lane), and transferred to polyvinylidene difluoride membranes. After Ponceau S staining, membranes were incubated with primary antibodies followed by horseradish peroxidase-conjugated secondary antibodies. Signals were developed with Western Lightning Plus-ECL (PerkinElmer, Shelton, CT, USA). Band intensities were quantified using ImageJ (version 1.53c, NIH, Bethesda, MD, USA). Protein expression was normalized to the total protein level on the membrane, as visualized by Ponceau S staining.^17^ Antibody information is provided in Table S4.

### 2.7 Immunofluorescence

Jejunal tissues were embedded in OCT compound (Sakura Finetek, Tokyo, Japan). Cryosections (7 μm) were fixed in 4% paraformaldehyde, permeabilized with 0.2% Triton X-100, and blocked with 5% normal goat or donkey serum. Sections were incubated with primary antibodies, followed by secondary antibodies and 4′,6-diamidino-2-phenylindole (DAPI). Antibody details are listed in Table S4. Fluorescence images were acquired using a Leica FW4000 microscope (Leica Microsystems, Wetzlar, Germany). For quantitative analysis, fluorescence intensity was measured using ImageJ software. The fluorescence intensity of each target protein was quantified within a defined tissue area and expressed as fluorescence intensity per unit area.

### 2.8 Fecal SCFA Analysis

SCFAs, including acetic, propionic, n-butyric, and n-valeric acids, were quantified using an established ultraperformance liquid chromatography–mass spectrometry method, as previously described.^18^ Briefly, fecal samples were homogenized in 9 volumes of ultrapure water, and the supernatants were collected. Proteins were precipitated with 70% acetonitrile on ice for 30 min. For derivatization, each standard or sample was mixed with 25 mM 3-nitrophenylhydrazine and 15 mM 1-ethyl-3-(3-(dimethylamino)­propyl)­carbodiimide in 50% acetonitrile containing 1.5% pyridine. Derivatized crotonic acid was added as an internal standard. The samples were then analyzed by ultraperformance liquid chromatography–mass spectrometry using an Acquity TQD system (Waters, Milford, MA, USA).

### 2.9 Microbiota Analysis (16S rRNA Gene Sequencing)

Bacterial DNA was extracted from cecal contents using the QuickGene DNA Tissue Kit (Kurabo, Osaka, Japan). The V3–V4 region of the 16S rRNA gene was amplified using primers 341F and 805R, and libraries were sequenced on an Illumina MiSeq platform.^19,20^ Sequence data were processed with QIIME2 (version 2024.5) using DADA2 for denoising. Taxonomy was assigned using a scikit-learn classifier trained on the SILVA 138 database (99% OTUs). Singletons and mitochondrial/chloroplast ASVs were removed. Phylogenetic trees were constructed using SATé-enabled phylogenetic placement. The 16S rRNA gene sequencing data generated in this study have been deposited in the NCBI Sequence Read Archive (SRA) under BioProject accession number PRJNA1454693.

### 2.10 Statistical Analysis

Data are expressed as mean ± s.e.m. For comparisons between two groups, Student’s *t* test or Mann–Whitney U test were used. For comparisons among three or more groups, one-way ANOVA followed by the Tukey–Kramer post hoc test was used for parametric data, whereas the Kruskal–Wallis test followed by the Steel–Dwass post hoc test was used for nonparametric data. A value of *P* < 0.05 was considered statistically significant.

## 3 Results

### 3.1 Soybean Fiber Enhances Antimicrobial Protein Expression in the Mouse Small Intestine

Whole-transcriptome analysis was conducted in the jejunum to identify genes affected by WSSF supplementation. A total of 414 genes were upregulated more than 2-fold compared with the control group, whereas 3023 genes were downregulated (Figure 1B). Gene Ontology (GO) enrichment analysis of the upregulated genes using Metascape^21^ revealed that the top enriched terms included immune-related pathways such as “humoral immune response” and “acute-phase response,” both of which are closely associated with antimicrobial defense (Figure 1C).

As shown in Figure 1D, WSSF increased the expression of several AMP genes, including *Retnlb*, *Sprr2a*, *Reg3*, and *Ang4*. qRT-PCR further confirmed that *Sprr2a*, *Retnlb*, and *Ang4* mRNA levels were dose-dependently upregulated in the jejunum (Figure 1E–G). These increases were reflected at the protein level, as determined by immunoblot analysis (Figure 1H–J, Figure S2). Immunofluorescence also demonstrated an increased number of RELMβ, SPRR2A, and ANG4-positive epithelial cells following WSSF feeding (Figure 1K, Figure S3). In contrast, the WSSF-induced increases in *Reg3b* and *Reg3g* were not reproducible by qRT-PCR (Figure S4A,B).

Similarly to the jejunum, WSSF increased RELMβ, SPRR2A, and ANG4 expression at both mRNA and protein levels in the ileum (Figure 2A–F, Figure S5). In the colon, however, no significant changes were observed in the expression of these AMPs (Figure S6A–C). Because dietary fiber is metabolized by intestinal microorganisms, particularly in the cecum and colon, we next measured fecal SCFAs. The levels of acetate, propionate, and n-butyrate, but not n-valerate, were significantly higher in the 10% WSSF group than in the control group (Figure S7). These observations suggest elevated SCFA in lower intestine are unlikely account for the AMP induction. Collectively, these findings demonstrate that WSSF robustly upregulates AMPs such as RELMβ, SPRR2A, and ANG4 in the mouse small intestine, possibly through direct interactions with epithelial cells.

**Figure 2:**
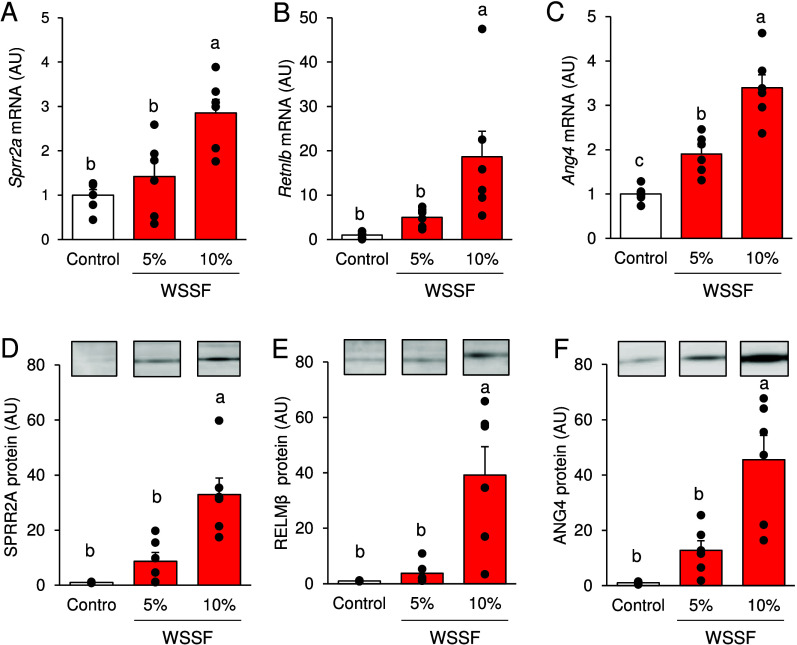
Soybean fiber enhances antimicrobial protein expression in the mouse ileum.Data shown are from Experiment 1. Mice were fed control diets or diets supplemented with 5% or 10% water-soluble soybean fiber (WSSF) for 5 days, after which ileal tissues were collected. mRNA expression levels of Sprr2a, Retnlb, and Ang4 were quantified by quantitative reverse transcription-PCR (qRT-PCR; A–C), and protein expression levels were evaluated by immunoblotting (D–F). Data are presented as mean ± s.e.m. Statistical significance was assessed using the Tukey–Kramer post hoc test or the Steel–Dwass test. Groups not sharing a common letter are significantly different (*p* < 0.05). AU: arbitrary unit.

### 3.2 Soybean Fiber Activates the Tuft Cell–ILC2-Axis Leading to IL-13 Production

Previous studies have shown that tuft cell–derived IL-25 activates ILC 2s to stimulate IL-13 production, which subsequently induces epithelial AMP expression and tuft cell expansion.^7^ Consistent with this pathway, WSSF supplementation increased the mRNA expression of *Il25* (Figure 3A,B), and tuft cell markers *Pou2f3* and *Dclk1* in both the jejunum and ileum (Figure 3C–F), although the increase in *Il25* in the jejunum did not reach statistical significance. Immunofluorescence analysis demonstrated an increased number of DCLK1-positive tuft cells following WSSF feeding (Figure 3G, Figure S3). WSSF also increased *Il13* mRNA expression in these regions (Figure 3H,I). To examine whether this response reflected basal regional differences in tuft cell-related gene expression, we compared jejunal and colonic tissues from control mice. Basal Il25 mRNA expression was higher in the colon than in the jejunum, whereas Pou2f3 and Trpm5 mRNA expression did not differ between these regions (Figure S8).

**Figure 3:**
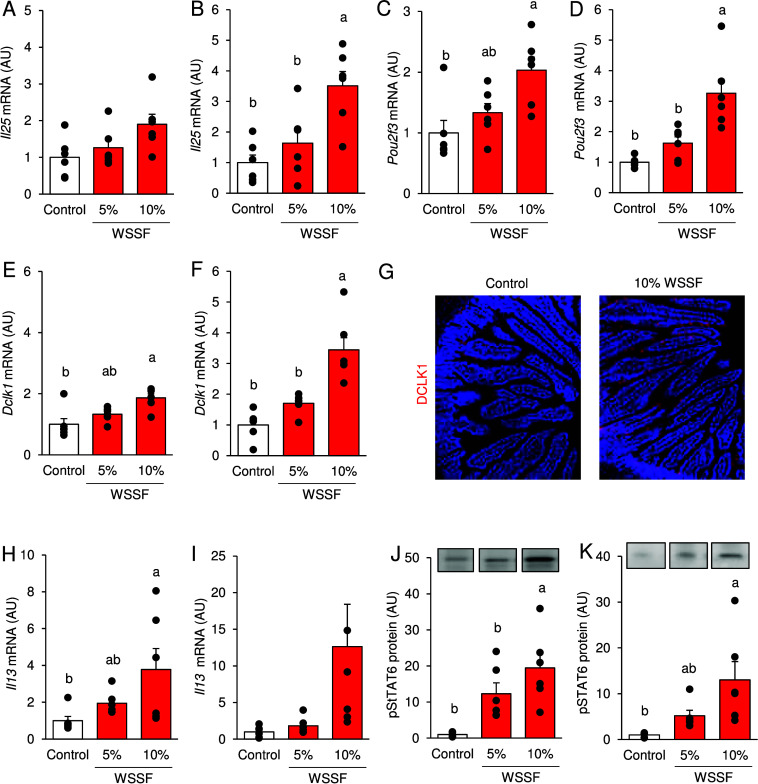
Soybean fiber upregulates tuft cell markers and type 2 immunity-related molecules in the mouse small intestine. Data shown are from Experiment 1. Mice were fed control diets or diets supplemented with 10% water-soluble soybean fiber (WSSF) for 5 days, after which jejunal (A, C, E, G, I) and ileal (B, D, F, H, J) tissues were collected. mRNA expression levels of *Il25* (A, B), *Pou2f3* (C, D), *Dclk1* (E, F), and *Il13* (G, H) were quantified by quantitative reverse transcription-PCR (qRT-PCR; A–H). Levels of STAT6 phosphorylation were evaluated by immunoblotting (I, J). Representative immunofluorescence images of DCLK1 in the jejunum are shown (K). Data are presented as mean ± s.e.m. Statistical significance was assessed using the Tukey–Kramer post hoc test or the Steel–Dwass test. Groups not sharing a common letter are significantly different (*p* < 0.05). AU: arbitrary unit.

IL-13 released from activated ILC 2s promotes STAT6 phosphorylation, which is required for inducing AMPs such as SPRR2A, RELMβ, and ANG4. Indeed, jejunal, and ileal, STAT6 phosphorylation increased in a dose-dependent manner following WSSF feeding (Figure 3J,K). Moreover, *Il25* and *Il13* expression levels positively correlated with AMPs (SPRR2A, RELMβ, ANG4) and tuft cell markers (*Pou2f3*, *Dclk1*) in both intestinal regions (Figure S9).

In contrast, *Il33*, an epithelial alarmin known to activate ILC 2s during tissue damage, showed no significant changes in the jejunum (Figure S10). These results indicate that WSSF increases AMP expression through activation of type 2 immunity and the tuft cell–ILC2 axis.

### 3.3 ILC2 Is Involved in Soybean Fiber-Mediated Antimicrobial Protein Expression

To examine whether ILC 2s are required for WSSF-mediated AMP induction, mice receiving WSSF-containing diets were treated with the ILC2 inhibitor disulfiram (Figure 4A). Disulfiram reduced WSSF-induced RELMβ, SPRR2A, and ANG4 expression at both the mRNA and protein levels in the jejunum (Figure 4B–G, Figure S11). In addition, disulfiram suppressed WSSF-induced increases in *Il25*, *Il13*, and tuft cell marker genes (*Dclk1* and *Pou2f3*) (Figure 5A–D). Furthermore, disulfiram attenuated WSSF-mediated STAT6 phosphorylation (Figure 5E, Figure S11). Collectively, these findings demonstrate that ILC2-mediated type 2 immunity is essential for WSSF-induced AMP expression in the small intestine.

**Figure 4:**
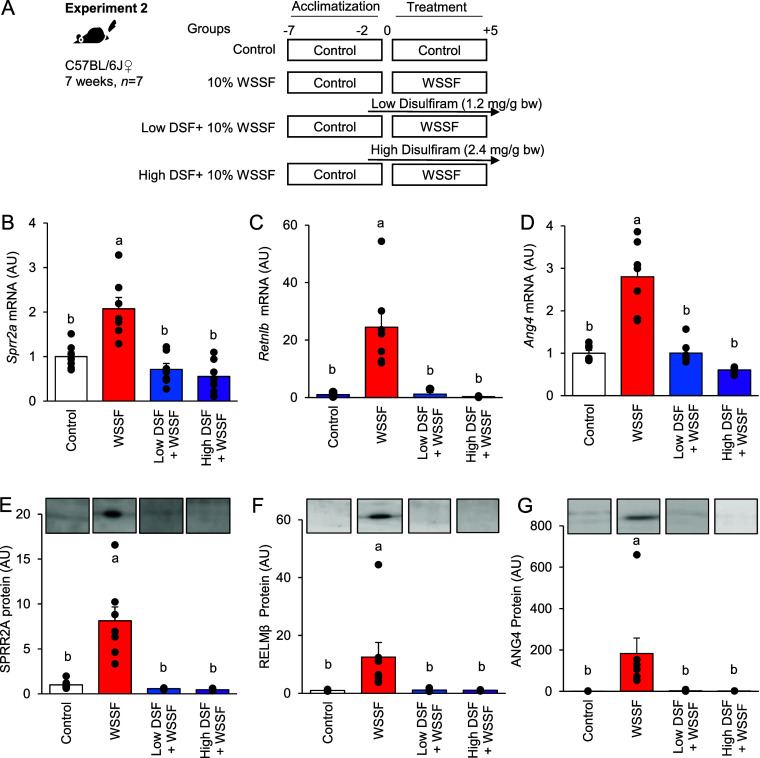
ILC2 is involved in soybean fiber-mediated antimicrobial protein expression in the mouse jejunum. Data shown are from Experiment 2. Mice were fed control diets or diets supplemented with 10% water-soluble soybean fiber (WSSF) for 5 days. Mice receiving the WSSF-containing diet were treated with the ILC2 inhibitor disulfiram (DSF) at two doses (low and high), after which jejunal tissues were collected, as illustrated in the experimental design (A). mRNA expression levels of *Sprr2a*, *Retnlb*, and *Ang4* were quantified by quantitative reverse transcription-PCR (qRT-PCR; B–D), and protein expression levels were evaluated by immunoblotting (E–G). Data are presented as mean ± s.e.m. Statistical significance was assessed using the Tukey–Kramer post hoc test or the Steel–Dwass test. Groups not sharing a common letter are significantly different (*p* < 0.05). AU: arbitrary unit.

**Figure 5:**
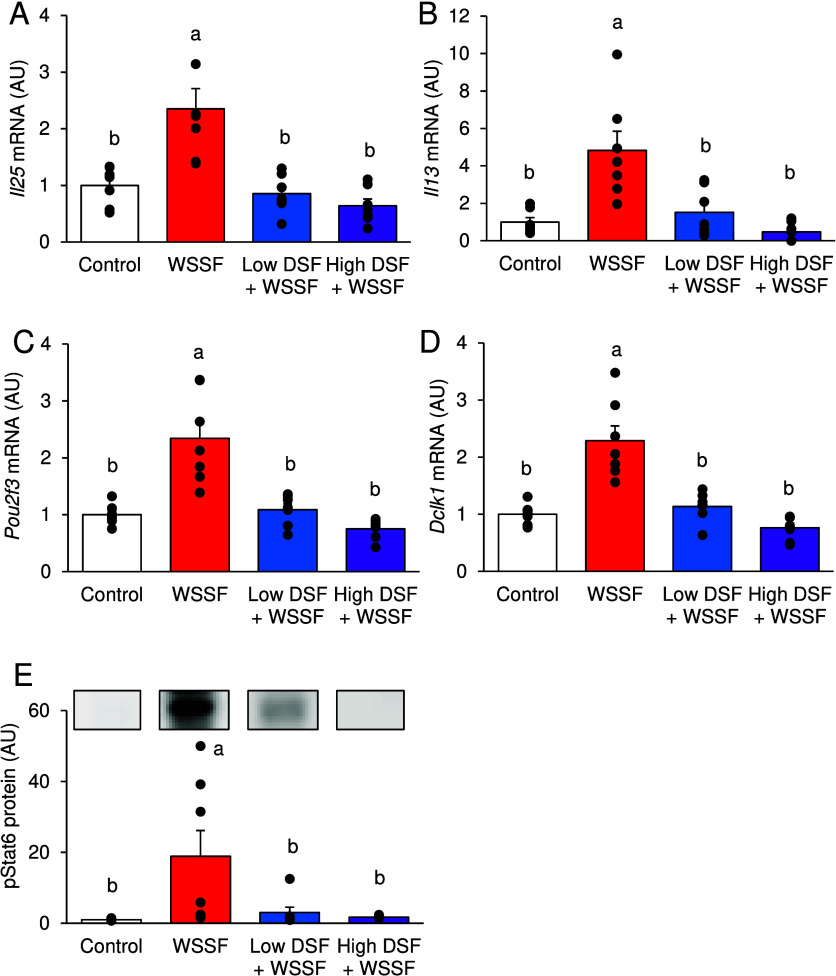
ILC2 is involved in soybean fiber-mediated upregulation of tuft cell markers and type 2 immunity-related molecules in the mouse jejunum. Data shown are from Experiment 2. Mice were fed control diets or diets supplemented with 10% water-soluble soybean fiber (WSSF) for 5 days. Mice receiving the WSSF-containing diet were treated with the ILC2 inhibitor disulfiram (DSF) at two doses (low and high), after which jejunal tissues were collected. mRNA expression levels of *Il25*, *Il13*, *Pou2f3,* and *Dclk1* were quantified by quantitative reverse transcription-PCR (qRT-PCR; A–D). Levels of STAT6 phosphorylation were evaluated by immunoblotting (E). Data are presented as mean ± s.e.m. Statistical significance was assessed using the Tukey–Kramer post hoc test or the Steel–Dwass test. Groups not sharing a common letter are significantly different (*p* < 0.05). AU: arbitrary unit.

### 3.4 TRPM5 Is Involved in Soybean Fiber-Mediated Antimicrobial Protein Expression

TRPM5, a monovalent cation channel highly expressed in tuft cells, plays a key role in IL-25 production.^22^ WSSF supplementation increased jejunal AMP expression, including RELMβ, SPRR2A, ANG4, at both mRNA and protein levels; however, these increases were attenuated by TPPO, a well-established TRPM5 inhibitor (Figure 6B–G, Figure S12). Immunofluorescence analysis further confirmed the inhibitory effects of TPPO on WSSF-induced AMP expression (Figure 6H, Figure S13). Although *Il25* expression was not significantly altered in this study (Figure 7A), TPPO reduced the WSSF-induced expression of *Pou2f3*, *Dclk1*, and *Il13*, as well as STAT6 phosphorylation (Figure 7B–E, Figure S12). Immunofluorescence analysis confirmed the inhibitory effect of TPPO on WSSF-induced expansion of DCLK1-positive tuft cells (Figure 7F, Figure S13). These results indicate that TRPM5 is required for WSSF-mediated activation of type 2 immunity and tuft cell function.

**Figure 6:**
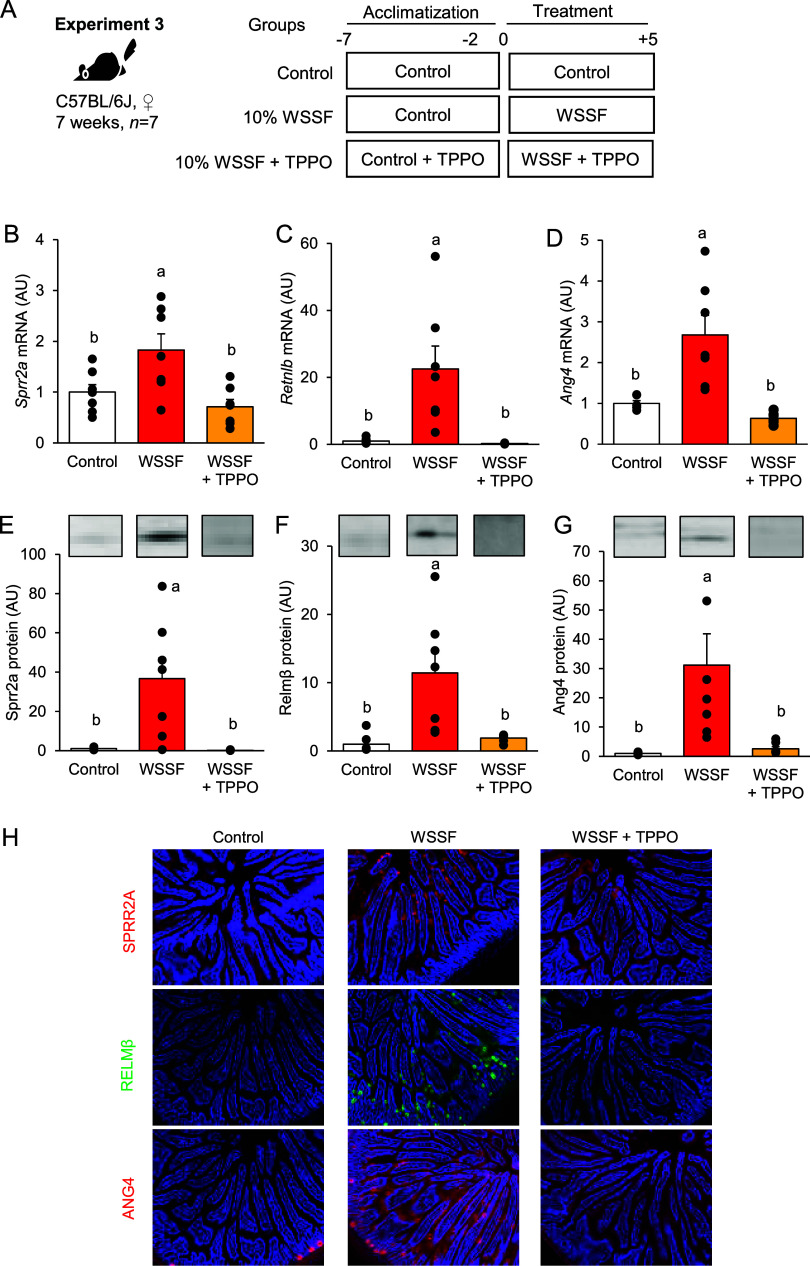
TRPM5 is involved in soybean fiber-mediated antimicrobial protein expression. Data shown are from Experiment 3. Mice were fed control diets or diets supplemented with 10% water-soluble soybean fiber (WSSF) for 5 days. Mice receiving the WSSF-containing diet were treated with the TRPM5 inhibitor triphenylphosphine oxide (TPPO), after which jejunal tissues were collected, as illustrated in the experimental design (A). mRNA expression levels of *Sprr2a*, *Retnlb*, and *Ang4* were quantified by quantitative reverse transcription-PCR (qRT-PCR; B–D), and protein expression levels were evaluated by immunoblotting (E–G). Representative immunofluorescence images of SPRR2A, RELMβ, and ANG4 in the jejunum are shown (H). Data are presented as mean ± s.e.m. Statistical significance was assessed using the Tukey–Kramer post hoc test or the Steel–Dwass test. Groups not sharing a common letter are significantly different (*p* < 0.05). AU: arbitrary unit.

**Figure 7:**
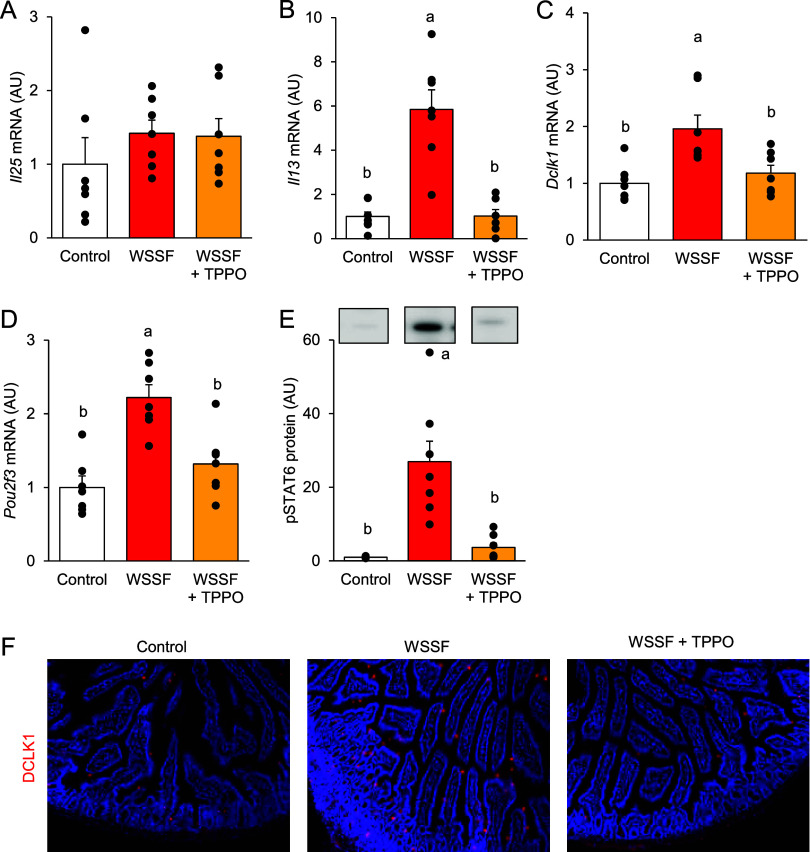
TRPM5 is involved in soybean fiber-mediated antimicrobial protein expression in the mouse jejunum. Data shown are from Experiment 3. Mice were fed control diets or diets supplemented with 10% water-soluble soybean fiber (WSSF) for 5 days. Mice receiving the WSSF-containing diet were treated with the TRPM5 inhibitor triphenylphosphine oxide (TPPO), after which jejunal tissues were collected. mRNA expression levels of *Il25*, *Il13*, *Dlck1,* and *Pou2f3* were quantified by quantitative reverse transcription-PCR (qRT-PCR; A–D). Levels of STAT6 phosphorylation were evaluated by immunoblotting (E). Representative immunofluorescence images of DCLK1 in the jejunum are shown (F). Data are presented as mean ± s.e.m. Statistical significance was assessed using the Tukey–Kramer post hoc test or the Steel–Dwass test. Groups not sharing a common letter are significantly different (*p* < 0.05). AU: arbitrary unit.

### 3.5 Tuft Cells Are Required for Soybean Fiber-Mediated Antimicrobial Protein Expression

Tuft cells regulate type 2 immunity and AMP expression by sensing luminal chemical cues.^23^ Consistent with previous findings, WSSF increased SPRR2A, RELMβ, and ANG4 expression in the jejunum of wild-type mice, as shown by qRT-PCR and immunoblotting (Figure 8B–G, Figure S14). In contrast, these increases were absent in tuft cell–deficient *Pou2f3*-KO mice, which was further confirmed by immunofluorescence (Figure 8H, Figure S15). WSSF-induced increases in *Il13*, *Il25*, tuft cell markers (*Pou2f3, Dclk1*), and STAT6 phosphorylation were also observed in wild-type but not *Pou2f3*-KO mice (Figure 9A–F, Figure S14). Similar trends were observed in the ileum, although the increases in *Sprr2a, Il13*, and *Dclk1* did not reach statistical significance (Figures S16A–I and S17). These findings indicate that tuft cells are required for WSSF-mediated AMP induction and type 2 immunity.

**Figure 8:**
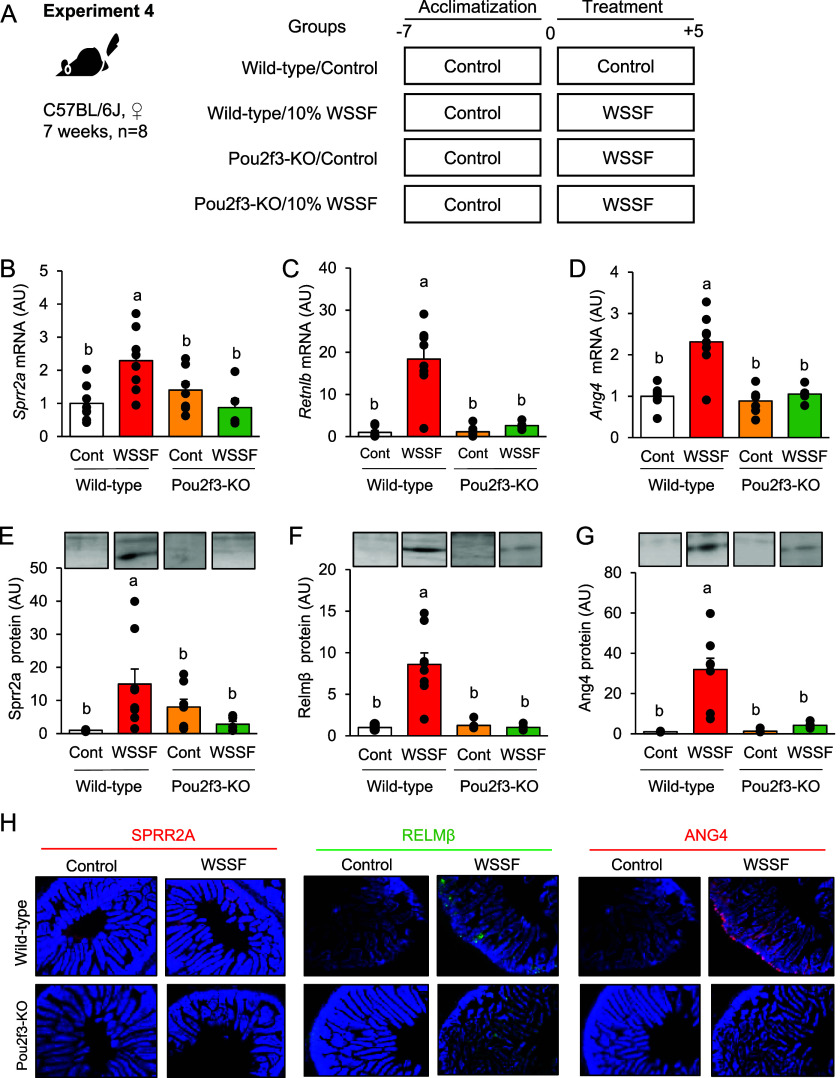
Tuft cells are required for soybean fiber-mediated antimicrobial protein expression. Data shown are from Experiment 4. *Pou2f3*-KO mice and wild-type littermates were fed control diets or diets supplemented with 10% water-soluble soybean fiber (WSSF) for 5 days, after which jejunal tissues were collected, as illustrated in the experimental design (A). mRNA expression levels of *Sprr2a*, *Retnlb*, and *Ang4* were quantified by quantitative reverse transcription-PCR (qRT-PCR; B–D), and protein expression levels were evaluated by immunoblotting (E–G). Representative immunofluorescence images of SPRR2A, RELMβ, and ANG4 in the jejunum are shown (H). Data are presented as mean ± s.e.m. Statistical significance was assessed using the Tukey–Kramer post hoc test or the Steel–Dwass test. Groups not sharing a common letter are significantly different (*p* < 0.05). AU: arbitrary unit; KO: knockout.

**Figure 9:**
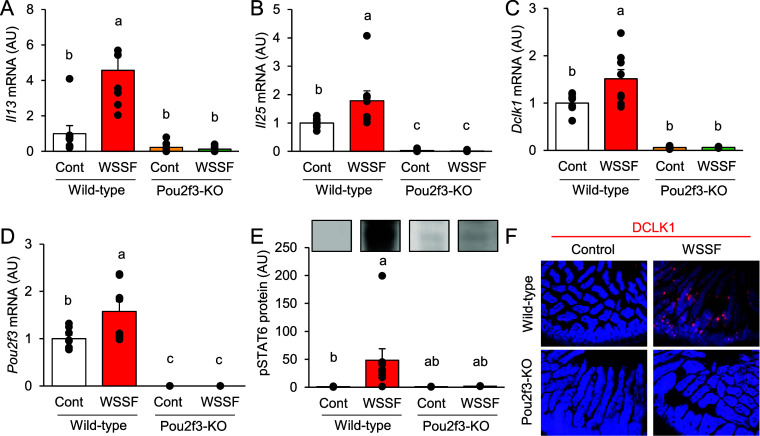
Tuft cells are required for soybean fiber-mediated upregulation of tuft cell markers and type 2 immunity-related molecules in the mouse jejunum. Data shown are from Experiment 4. *Pou2f3*-KO mice and wild-type littermates were fed control diets or diets supplemented with 10% water-soluble soybean fiber (WSSF) for 5 days, after which jejunal tissues were collected. mRNA expression levels of *Il25*, *Il13*, *Pou2f3*, and *Dclk1* were quantified by quantitative reverse transcription-PCR (qRT-PCR; B–D). Levels of STAT6 phosphorylation were evaluated by immunoblotting (E). Representative immunofluorescence images of DClK1 in the jejunum are shown (F). Data are presented as mean ± s.e.m. Statistical significance was assessed using the Tukey–Kramer post hoc test or the Steel–Dwass test. Groups not sharing a common letter are significantly different (*p* < 0.05). AU: arbitrary unit; KO: knockout.

### 3.6 Soybean Fiber Supplementation Alters Cecal Microbiota Composition

AMPs contribute not only to protection against pathogens but also to maintaining steady-state microbial composition.^24^ To investigate whether WSSF alters gut microbiota, we analyzed cecal microbiota, which is anatomically more relevant to small-intestinal AMP activity than colon or feces. WSSF reduced microbial diversity, as assessed by observed species counts and the Chao1 index, in *Pou2f3*-KO but not wild-type mice (Figure 10A,B). Shannon diversity was also reduced by WSSF only in *Pou2f3*-KO mice (Figure 10C). Principal coordinate analysis based on Bray–Curtis, weighted UniFrac, and unweighted UniFrac distances showed that WSSF influenced β-diversity (Figure 10D). Genotype-dependent effects were evident only in unweighted UniFrac analysis, which is more sensitive to rare taxa.

**Figure 10:**
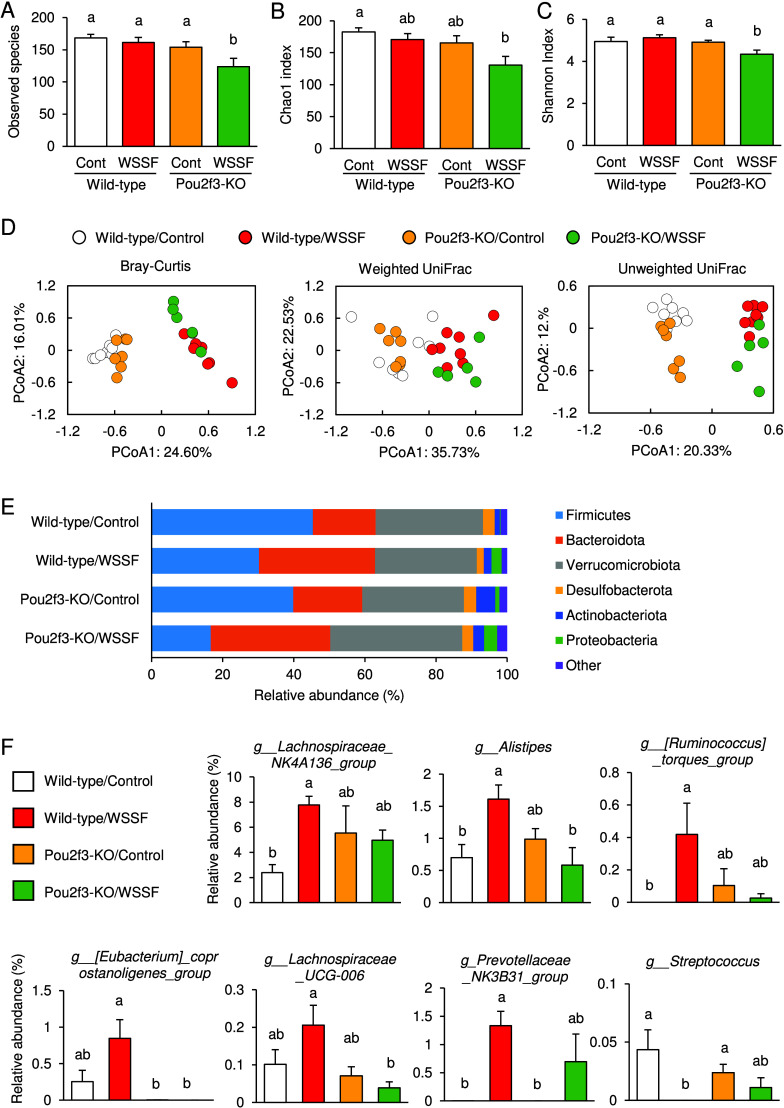
Soybean fiber supplementation alters cecal microbiota composition. Data shown are from Experiment 4. *Pou2f3*-KO mice and wild-type littermates were fed control diets or diets supplemented with 10% water-soluble soybean fiber (WSSF) for 5 days, after which cecal contents were collected. Cecal microbiota composition was analyzed by 16S rRNA gene sequencing, and sequence data were processed using QIIME 2. Alpha diversity metrics, including the number of observed species (A), Chao1 index (B), and Shannon evenness (C), were calculated. Principal coordinate analysis plots of the cecal microbiota were generated based on Bray–Curtis, weighted UniFrac, and unweighted UniFrac distances (D). Mean relative abundances of cecal microbiota at the phylum level are shown (E), and relative abundances of selected taxa at the genus level are shown (F). Data are presented as mean ± s.e.m. Statistical significance was assessed using the Tukey–Kramer post hoc test or the Steel–Dwass test. Groups not sharing a common letter are significantly different (*p* < 0.05). KO: knockout.

Six phyla—Bacteroidota, Firmicutes, Actinobacteriota, Proteobacteria, Desulfobacterota, and Verrucomicrobiota—dominated the microbiota (Figure 10E). The effects of WSSF, genotype, and their interaction were evaluated by two-way ANOVA (Figure S18). Two-way ANOVA revealed that WSSF affected Bacteroidota, Firmicutes, and Proteobacteria, increasing the former two and decreasing Firmicutes. *Pou2f3*-KO genotype affected Firmicutes, Actinobacteriota, and Proteobacteria, with Actinobacteriota and Proteobacteria enriched and Firmicutes reduced.

Several genera were significantly influenced by WSSF or genotype (Figure S19). Among abundant genera, WSSF decreased unclassified *Lachnospiraceae*, *Dubosiella*, *Colidextribacter*, *Blautia*, and uncultured *Oscillospiraceae*, while increasing *Bacteroides*, *Parasutterella*, *Bifidobacterium*, and *Prevotellaceae_NK3B31_group* (Figure S20). *Pou2f3*-KO mice exhibited lower levels of unclassified *Lachnospiraceae*, *UCG-010*, *Candidatus Saccharimonas*, and *Rikenellaceae_RC9_gut_group*, but higher levels of *Lactobacillus* and *Parasutterella* (Figure S20). Because WSSF upregulates intestinal AMPs in a tuft cell–dependent manner, we hypothesized that bacterial genera regulated by these AMPs would exhibit a significant WSSF × genotype interaction. Two-way ANOVA revealed significant interactions between WSSF and genotype in several genera, including *Lachnospiraceae_NK4A136_group*, *UCG-010*, *Candidatus Saccharimonas*, *Lactobacillus*, *Rikenellaceae_RC9_gut_group*, and *Alistipes* (Figure S19). Notably, WSSF increased or tended to increase *Lachnospiraceae_NK4A136_group*, *Alistipes*, *[Ruminococcus]_torques_group*, and *[Eubacterium]_coprostanoligenes_group* in wild-type mice, but not in *Pou2f3*-KO mice. In parallel, Pearson correlation analysis identified 30 genera whose abundances correlated with jejunal *Sprr2a*, *Retnlb*, or *Ang4* expression (Figure S21). Among these, WSSF selectively increased *Lachnospiraceae_UCG-006* and *Prevotellaceae_NK3B31_group*, as well as *[Ruminococcus]_torques_group* and *[Eubacterium]_coprostanoligenes_group*, specifically in wild-type mice, whereas *Streptococcus* was reduced only in wild-type mice (Figure 10F). Collectively, these data suggest that WSSF modulates cecal microbiota composition, at least in part, through tuft cell–dependent regulation of antimicrobial protein expression.

## 4 Discussion

The present study demonstrates that the soybean-derived fiber WSSF markedly upregulates the AMPs such as Sprr2a, RELMβ, and Ang4 in the mouse small intestine. We further showed that this response requires TRPM5, tuft cells, and ILC2–IL-13–STAT6 signaling, indicating that WSSF activates a tuft cell–ILC2 axis to enhance epithelial barrier defense. In addition, WSSF altered the composition of the cecal microbiota in a manner that partly depended on tuft cells and AMP expression. These findings suggest that WSSF not only serves as a fermentable dietary fiber but also functions as an epithelial–immune modulator that may contribute to intestinal health and disease prevention.

AMPs produced by intestinal epithelial cells are key effectors of innate defense and barrier integrity.^25^ SPRR2A, RELMβ, and ANG4 are predominantly expressed by Paneth and goblet cells and act at the mucosal surface to restrict bacterial contact with the epithelium and to shape microbial composition. Genetic and experimental studies have shown that reduced expression of these AMPs increases susceptibility to intestinal inflammation and infection. SPRR2A deficiency predisposes mice to bacterial encroachment, impaired barrier function, and colitis.^24^ RELMβ is required to maintain spatial segregation between bacteria and the epithelium and is important for host defense against enteric pathogens and helminths.^26^ Ang4, originally identified as a Paneth cell–derived ribonuclease-type AMP, exhibits broad antibacterial and antifungal activity and contributes to mucosal homeostasis.^27,28^ Therefore, the marked upregulation of SPRR2A, RELMβ, and ANG4 by WSSF in the small intestine suggests a mechanism by which soybean-derived fiber strengthens the intestinal barrier defense. These findings also suggest potential relevance of WSSF to disease settings characterized by impaired barrier function and defective antimicrobial defense, including inflammatory bowel disease and enteric infection. Although the present study was conducted in healthy mice, our results provide a rationale for future studies examining whether WSSF exerts protective effects in disease models associated with mucosal inflammation and microbial challenge.

Our mechanistic data indicate that WSSF engages the tuft cell–IL-25–ILC2–IL-13 axis to induce AMP expression. Tuft cells are chemosensory epithelial cells that sense luminal cues and produce IL-25, which activates ILC2 to secrete IL-13.^29,30^ IL-13 then acts on epithelial cells to promote tuft and goblet cell expansion and to induce AMP expression via STAT6-dependent transcription.^23^ Consistent with this model, WSSF increased the expression of tuft cell markers (POU2F3 and DCLK1), type2-related cytokines (IL-25 and IL-13), and STAT6 phosphorylation in the small intestine. Pharmacological inhibition of TRPM5, a cation channel required for tuft cell chemosensory signaling and IL-25 production,^16,31^ with TPPO attenuated WSSF-induced increases in AMPs, tuft cell markers, IL-13, and STAT6 phosphorylation. Furthermore, disulfiram, used here as an ILC2 inhibitor,^15^ reduced WSSF-induced AMP expression and suppressed IL-25, IL-13, tuft cell markers, and STAT6 phosphorylation. Most importantly, WSSF failed to increase AMPs, tuft cell markers, IL-25, IL-13, or STAT6 phosphorylation in tuft cell–deficient *Pou2f3*-KO mice. Together, these results support a model in which WSSF is sensed by TRPM5-dependent tuft cells, leading to IL-25 production, ILC2 activation, IL-13 secretion, and subsequent STAT6-mediated induction of epithelial AMPs.

Previous studies have shown that several dietary fibers improve intestinal barrier function, but the reported mechanisms vary depending on fiber type. Fermentable fibers are often discussed in the context of microbiota-dependent effects, including increased SCFA production, enhanced mucus secretion, reinforcement of tight junctions, and suppression of inflammatory responses.^1,2^ In contrast, our findings suggest that WSSF exerts a distinct effect by strongly inducing the epithelial AMPs SPRR2A, RELMβ, and ANG4 in the small intestine through a tuft cell–IL-25–ILC2–IL-13–STAT6 pathway. This mechanism shares some similarity with our previous observations for psyllium, which also promoted type 2 immune responses via tuft cells,^15^ but the present study extends this concept to soybean-derived soluble fiber and demonstrates its requirement for TRPM5-dependent tuft cell signaling and Pou2f3-positive tuft cells. Thus, although barrier-protective effects appear to be a common feature of multiple dietary fibers, future studies are needed to determine whether different fiber types induce AMPs through shared tuft cell–dependent pathways or through distinct fiber-specific mechanisms.

Tuft cells have recently been recognized as intestinal sentinels that sense luminal cues to initiate type 2 mucosal immunity.^32,33^ Previous work has mainly focused on microbe- and parasite-derived metabolites, such as succinate and N-undecanoylglycine, which activate tuft cells in a manner dependent on receptors such as the succinate receptor SUCNR1, the olfactory receptor Vmn2r26, and bitter taste receptors of the Tas2R family.^9,34,35^ Our findings extend this concept by showing that a defined dietary fiber can also activate tuft cells and thereby drive AMP production. Because dietary fibers, including WSSF, do not permeate the plasma membrane of tuft cells, WSSF is likely sensed by tuft cells through specific, yet unidentified, receptors. Previous studies have demonstrated that dietary fibers such as inulin, pectin, and guar gum regulate the intestinal barrier and inflammation through Toll-like receptors and dectins.^36–38^ Further studies are required to clarify the molecular mechanisms by which intestinal tuft cells sense WSSF.

We also observed that WSSF modified the cecal microbiota composition, with several changes depending at least in part on tuft cells and AMP induction. AMPs not only kill pathogens but also help maintain a balanced microbiota by limiting bacterial access to the epithelium and selectively restricting susceptible taxa.^5^ Notably, WSSF increased or tended to increase genera such as *Alistipes*, *Lachnospiraceae_UCG-006*, *Lachnospiraceae_NK4A136_group*, *Prevotellaceae_NK3B31_group*, *[Ruminococcus]_torques_group*, and *[Eubacterium]_coprostanoligenes_group* in wild-type but not *Pou2f3*-KO mice. Many of these taxa are associated with beneficial functions; for example, *Alistipes* can metabolize tryptophan into indole derivatives that strengthen the intestinal barrier, *Lachnospiraceae* genera are often involved in short-chain fatty acid production, and the *[Eubacterium]_coprostanoligenes_group* is involved in cholesterol-to-coprostanol conversion, which may improve lipid metabolism.^39–41^ These observations support the idea that WSSF shapes the microbiota at least in part through tuft cell–dependent AMP induction. In parallel, supplemental WSSF also has a marked impact on the cecal microbiota through direct fiber–microbe interactions. Many genera, including *Bacteroides*, *Dubosiella*, *Colidextribacter*, and *Bifidobacterium*, were similarly influenced by WSSF in both wild-type and *Pou2f3*-KO mice. Together, our findings shed new light on the molecular mechanisms underlying dietary fiber-mediated changes in microbiota composition. Interestingly, WSSF reduced α-diversity only in *Pou2f3*-KO mice. One possible explanation is that, in the absence of tuft cell-dependent epithelial responses, WSSF may exert a more selective effect on bacterial growth through direct fiber utilization, thereby reducing overall community evenness. In wild-type mice, by contrast, tuft cell-dependent AMP induction and related epithelial responses may help maintain microbial balance despite WSSF supplementation. Because this interpretation remains speculative, further studies will be needed to determine how tuft cell-dependent epithelial programs influence microbiota diversity in response to WSSF.

Notably, WSSF induced type 2 immune activation and upregulated AMPs in the small intestine, but not in the colon. Because the colon harbors a much denser microbial community and is a major site of dietary fiber fermentation, this regional difference suggests that increased fecal SCFAs alone are unlikely to fully account for WSSF-induced AMP expression in the small intestine. Several factors may explain the lack of responsiveness in the colon. First, the colon contains a well-defined mucus layer, approaching 1 mm in thickness, whereas the mucus layer in the small intestine is discontinuous and less organized.^42^ This thick colonic mucus barrier may limit the access of WSSF to epithelial tuft cells. Second, once WSSF reaches the cecum and colon, it is likely to be rapidly degraded by resident microbiota, as suggested by the increased production of SCFAs. Such structural degradation may reduce the ability of WSSF to stimulate tuft cells. In addition, colonic tuft cells may differ from small-intestinal tuft cells in their sensory properties toward WSSF. Although we did not observe marked regional differences in *Pou2f3* or *Trpm5* expression between the small intestine and colon, basal *Il25* expression was higher in the colon than in the small intestine. Thus, differences in basal expression of these tuft cell-related genes do not appear to fully explain the region-specific AMP response observed in the present study. Recent studies have shown that tuft cells exhibit region-specific transcriptomic profiles and comprise distinct subtypes, such as tuft-1 and tuft-2.^43,44^ Supporting this idea, succinate activates tuft cells through SUCNR1 and induces type 2 immune responses in the small intestine, but not in the colon, where SUCNR1 expression is absent or minimal.^43^ Further studies will be needed to identify the mechanisms underlying the regional specificity of WSSF-mediated epithelial responses.

From a translational perspective, several issues should be addressed before WSSF can be developed as a functional food ingredient for intestinal barrier support. Soluble fiber structure can be modified during food processing, including by heating, pH changes, and mechanical treatment, and such changes may alter biological activity. Thus, future studies should determine whether processing-induced structural changes in WSSF affect its ability to regulate tuft cell–ILC2 signaling and AMP induction. In addition, the 5% WSSF diet used in this study corresponds to approximately 12.6 g fiber/1,000 kcal based on the energy density of the AIN-93G diet, a level broadly comparable to the U.S. reference value for total dietary fiber intake (14 g/1,000 kcal).^45^ However, direct translation of these mouse dietary inclusion levels to practical human intake is not straightforward, and the 10% diet should be interpreted as a relatively high experimental condition for defining biological efficacy. WSSF may also be useful in combination with other functional food factors, such as probiotics or polyphenols, to provide complementary benefits for intestinal barrier regulation. These topics warrant further investigation for translational application and food design.

The present study has several limitations. First, all experiments were conducted in mice over a short feeding period, and the duration of WSSF supplementation may not reflect typical human intake. The long-term effects of WSSF on intestinal antimicrobial protein expression, epithelial–immune interactions, and microbial adaptation remain to be determined. Second, the involvement of ILC 2s, TRPM5, and downstream STAT6 signaling was evaluated mainly using pharmacological inhibitors and associated molecular changes. Because disulfiram and TPPO may exert off-target effects and are not fully specific, the present results should be interpreted with caution. Moreover, although WSSF increased STAT6 phosphorylation together with AMP expression, this study did not directly determine whether STAT6 is required for WSSF-induced AMP upregulation. Therefore, while our findings support the involvement of the TRPM5–tuft cell–ILC2 axis and implicate STAT6 as a downstream mediator, further validation using genetic approaches, including ILC2-, TRPM5-, and epithelial Stat6-deficient mice, will be needed. Third, although our data implicate tuft cells in WSSF-induced epithelial–immune responses, the specific receptors or chemosensory mechanisms by which WSSF or its components are detected remain unknown. Tuft cells are known to sense luminal cues, including microbial metabolites and parasite-derived signals, through chemosensory pathways involving molecules such as TRPM5. However, whether WSSF directly activates tuft cells or indirectly modulates tuft cell activity through microbiota-derived metabolites or other epithelial–immune signals remains to be determined. Fourth, we did not directly distinguish between microbiota-independent and microbiota-dependent contributions of WSSF; germ-free or antibiotic-treated models will be required to precisely dissect these components. Finally, 16S rRNA–based profiling provides limited taxonomic and functional resolution, and future metagenomic or metabolomic analyses would help link AMP induction to specific microbial functions and metabolites.

In conclusion, our findings demonstrate that WSSF enhances the expression of the antimicrobial proteins SPRR2A, RELMβ, and Ang4 in the mouse small intestine through activation of a TRPM5-dependent tuft cell–ILC2–IL-13–STAT6 axis. This AMP induction is associated with changes in cecal microbiota composition that may favor barrier-supporting and metabolically beneficial taxa. By strengthening epithelial innate defense and modulating host–microbe interactions, WSSF may contribute to the maintenance of intestinal homeostasis and the prevention of inflammation-related diseases. These results highlight soybean-derived water-soluble fiber as a promising functional ingredient for targeting epithelial–immune pathways in the gut and warrant further investigation in human studies and mechanistic models.

## Supplementary Material



## Notes

(Figure S1) PCR genotyping of Pou2f3-knockout mice using acrylamide gel electrophoresis; (Figure S2) uncropped original immunoblot images corresponding to the blots shown in Figures 1 and 3; (Figure S3) quantification of SPRR2A, RELMβ, ANG4, and DCLK1 fluorescence intensity in the mouse jejunum from Figures 1K and 3G; (Figure S4) soybean fiber does not alter Reg3 gene expression in the mouse jejunum; (Figure S5) uncropped original immunoblot images corresponding to the blots shown in Figures 2 and 3; (Figure S6) soybean fiber does not alter gene expression of antimicrobial proteins and tuft cells-related markers in the mouse colon; (Figure S7) soybean fiber increases short-chain fatty acids in the mouse feces; (Figure S8) expression of tuft cell-related genes in the mouse jejunum and colon; (Figure S9) Pearson correlation analysis of mRNA expression of antimicrobial proteins, tuft cell markers, and type 2 cytokines in the mouse jejunum and ileum; (Figure S10) soybean fiber does not alter *Il33* gene expression in the mouse jejunum; (Figure S11) uncropped original immunoblot images corresponding to the blots shown in Figures 4 and 5; (Figure S12) uncropped original immunoblot images corresponding to the blots shown in Figures 6 and 7; (Figure S13) quantification of SPRR2A, RELMβ, ANG4, and DCLK1 fluorescence intensity in the mouse jejunum from Figures 6H and 7F; (Figure S14) uncropped original immunoblot images corresponding to the blots shown in Figures 8 and 9; (Figure S15) quantification of SPRR2A, RELMβ, ANG4, and DCLK1 fluorescence intensity in the mouse jejunum from Figures 8H and 9F; (Figure S16) tuft cells are required for soybean fiber-mediated antimicrobial protein expression and upregulation of tuft cell markers and type 2 immunity-related molecules in the mouse ileum; (Figure S17) uncropped original immunoblot images corresponding to the blots shown in Figure S16; (Figure S18) two-way ANOVA of phylum-level abundances in the cecal microbiota of mice fed soybean fiber; (Figure S19) two-way ANOVA of genus-level abundances in the cecal microbiota of mice fed soybean fiber; (Figure S20) microbiota composition at the genus level in cecal contents of mice fed soybean fiber; (Figure S21) Pearson correlation analysis between jejunal antimicrobial protein gene expression and cecal microbiota composition; (Table S1) primer sequences used for genotyping of Pou2f3-knockout mice; (Table S2) diet compositions in Experiments 1–4; (Table S3) primers for qRT-PCR analysis used in this study; (Table S4) details of antibodies used in this study (PDF)

## Abbreviations Used

AMP: antimicrobial protein
ANG4: angiogenin-4
ANOVA: analysis of variance
ASV: amplicon sequence variant
BCA: bicinchoninic acid
DAPI: 4′,6-diamidino-2-phenylindole
DCLK1: doublecortin-like kinase 1
DSF: disulfiram
GO: Gene Ontology
IL: interleukin
ILC2: group 2 innate lymphoid cell
KO: knockout
qRT-PCR: quantitative reverse transcription–polymerase chain reaction
RELMβ: resistin-like molecule beta
RNA-seq: RNA sequencing
SCFA: short-chain fatty acid
STAT6: signal transducer and activator of transcription 6
TPM: transcripts per million
TPPO: triphenylphosphine oxide
TRPM5: transient receptor potential cation channel subfamily M member 5
WSSF: water-soluble soybean fiber
