# Supplementary material for: Soybean-Derived Soluble Fiber Enhances Antimicrobial Proteins in the Mouse Small Intestine via a Tuft Cell–Group 2 Innate Lymphoid Cell Axis
Source: J Agric Food Chem. 2026 Jun 3;74(23):17952–67. doi: 10.1021/acs.jafc.6c01854 (PMC13281540; doi:10.1021/acs.jafc.6c01854)
Supplement: Supplementary file 1 [file jf6c01854_si_001.pdf]

Supporting Information

Soybean-Derived Soluble Fiber Enhances Antimicrobial Proteins in the Mouse Small Intestine via a Tuft Cell-ILC2 Axis

Arslan Ahmad[1], Bambang Dwi Wijatniko[1, 2], Cláudia Mapure Siueia[1], Chisato Yanagi[1], Yoshiki Ishii[1], Shodai Ishikawa[3] Ryo Inoue[3], Dina Mustika Rini[1], Noriyuki Yanaka[1], Takuya Suzuki[1*]

[1]Graduate School of Integrated Sciences for Life, Hiroshima University, 1-4-4 Kagamiyama, Higashi-Hiroshima 739-8528, Japan

[2]Department of Food and Agricultural Product Technology, Universitas Gadjah Mada, Yogyakarta, 55281, Indonesia

[3]Faculty of Agriculture, Setsunan University, 45-1 Nagaotoge-cho, Hirakata 573-0101 Japan

*Corresponding author:

E-mail: takuya@hiroshima-u.ac.jp

Tel: +81-82-424-7984, Fax: +81-82-424-7916

Table S1. Primer sequences used for genotyping of *Pou2f3*-knockout mice

Primer name	Forward (5′ to 3′)
Pou2f3-s	CCAAGATCTGGACTGCACTTCTTC
Skn-int7as	ATCTGCGCACAGCCTTGTATCTTC
Neo	GCAATCCATCTTGTTCAATGGCC

Table S2. Diet compositions in Experiments 1–4

Ingredients	Control	5% WSSF	10% WSSF
	g/kg diet		
Starch[1]	579.5	529.5	479.5
Sucrose[2]	100.0	100.0	100.0
Casein[3]	200.0	200.0	200.0
L-cysteine	3.0	3.0	3.0
Soybean oil	70.0	70.0	70.0
Mineral mix[4]	35.0	35.0	35.0
Vitamin mix[4]	10.0	10.0	10.0
Choline bitrate	2.5	2.5	2.5
Soybean fiber (WSSF)[5]	0.0	50.0	100.0
Total	1000.0	1000.0	1000.0

[1] Starch (Chuo-syokuryou Industry, Inazawa, Japan).

[2] Sucrose (Nissin Sugar, Tokyo, Japan).

[3] Casein (ALACID; New Zealand Daily Board, Wellington, New Zealand).

[4] Mineral and vitamin mixtures were prepared according to the AIN-93G formulation.

[5] SOYAFIBE-SDA100 (Fuji Foundation for Protein Research)

Table S3. Primers for qRT-PCR analysis used in this study

Target genes	Forward (5′ to 3′)	Reverse (5′ to 3′)
Mouse *Sprr2a*	TCTTCCTTCAGTGTGGCCTG	CACAGGAGGGCATGTTGACT
Mouse *Retnlb*	TCTCAGTCGTCAAGAGCCTAA	GCCACAAGCACATCCAGTGA
Mouse *Ang4*	ACCACTTGTACGCACTCAGG	TTAAAGGCTCGGTACCCGCA
Mouse *Il13*	CACACAAGACCAGACTCCCC	GTTGGTCAGGGAATCCAGGG
Mouse *Il25*	CCTTGGAGCTATGAGTTGGACA	TGTGGGAGCCTGTCTGTAGG
Mouse *Pou2f3*	TGTGCAAACTCAAGCCACTG	TCTTCCTGCCAAACACTTCG
Mouse *Dclk1*	AGCTGTCAGTAGCTGGCAAA	TCAAGAGCGGTGGTTGCTAT
Mouse *Il33*	ATTTCCCCGGCAAAGTTCAG	AGAACGGAGTCTCATGCAGTAG
Mouse *Trpm5*	ACAGAACATTGCTGCACACC	TTGCAGGCTTTCACAAGTGC
Mouse *Rps28*	ATCAAGCTGGCTAGGGTAACC	GGCCTTTGACATTTCGGATGA

Table S4. Details of antibodies used in this study

Antibodies	Source	Catalog Number	Immunoblot	Immunofluoresce nce
Rabbit anti-SPRR2A	Sigma-Aldrich	Custom-made	1:4000	1:200
Goat anti-RELMB	R & D System	AF2730	1:2000	1:200
Rabbit anti-ANG4	CUSABIO Tech.	PA661010ZA01MO	1:10000	1:600
Rabbit anti pSTAT6	Cell Signaling Tech.	56554	1:6000	1:200
Rabbit anti- DCLK1	Abcam	ab31704	---	1:1000
Goat anti-rabbit IgG-HRP	SeraCare	074-1506	1:20000	---
Goat anti-mouse IgG-HRP	SeraCare	074-1806	1:20000	---
Goat anti-rabbit IgG-AlexaFluor488	Abcam	ab150077	---	1:200
Donkey anti-goat IgG-AlexaFluor488	Abcam	ab150133	---	1:200

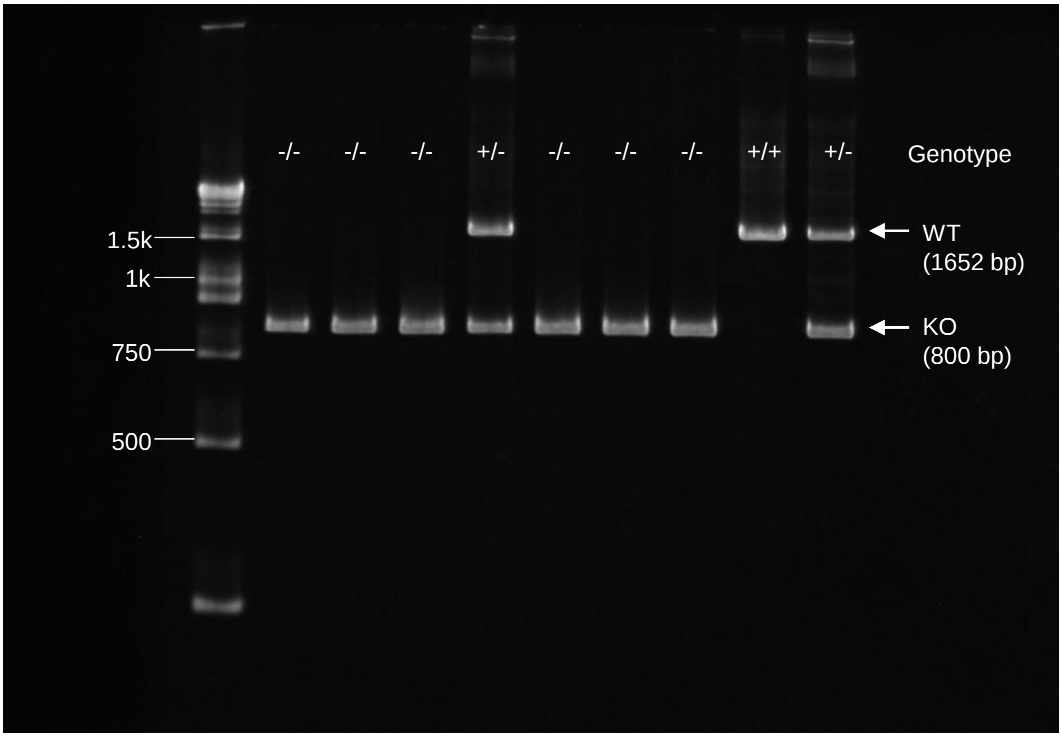

Figure S1. PCR genotyping of *Pou2f3*-knockout mice using acrylamide gel electrophoresis.

Homozygous knockout (KO) mice and wild-type littermates were generated by breeding heterozygous mice. Mouse genomic DNA was prepared from tail biopsies by an alkaline extraction method. Genotyping was carried out by PCR.

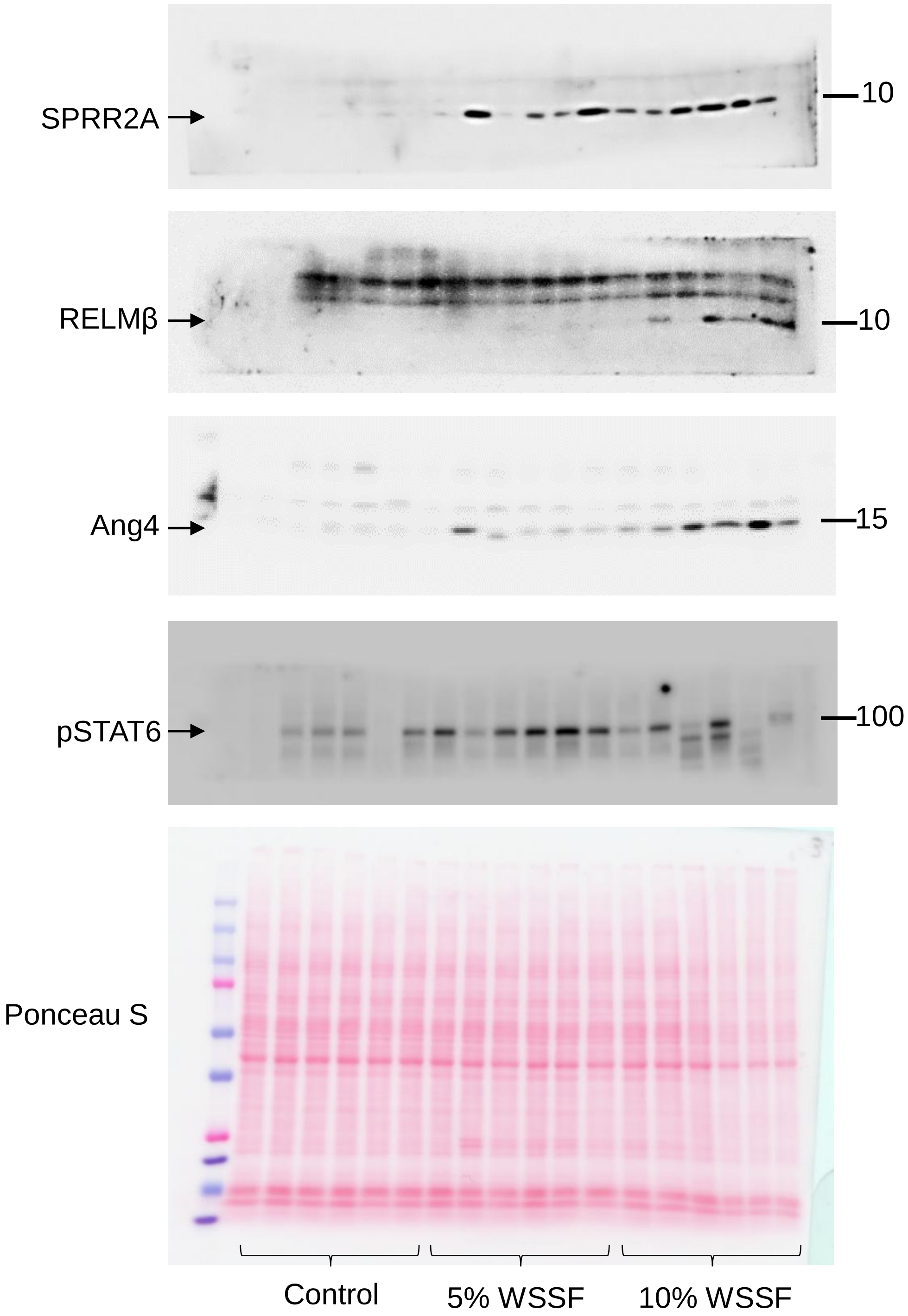

Figure S2. Uncropped original immunoblot images corresponding to the blots shown in Fig. 1 and 3.
Data are from Experiment 1. Mice were fed control diets or diets supplemented with 5% or 10% water-soluble soybean fiber (WSSF) for 5 days. Jejunal expression of SPRR2A, RELMβ, ANG4, and pSTAT6 was analyzed by immunoblotting. After transfer to PVDF membranes, total protein loading was visualized by Ponceau S staining and used for normalization. Membranes were cut horizontally according to molecular weight markers and probed separately with primary antibodies. The sample marked with an asterisk is unrelated to the present experiment and was not included in the analysis.

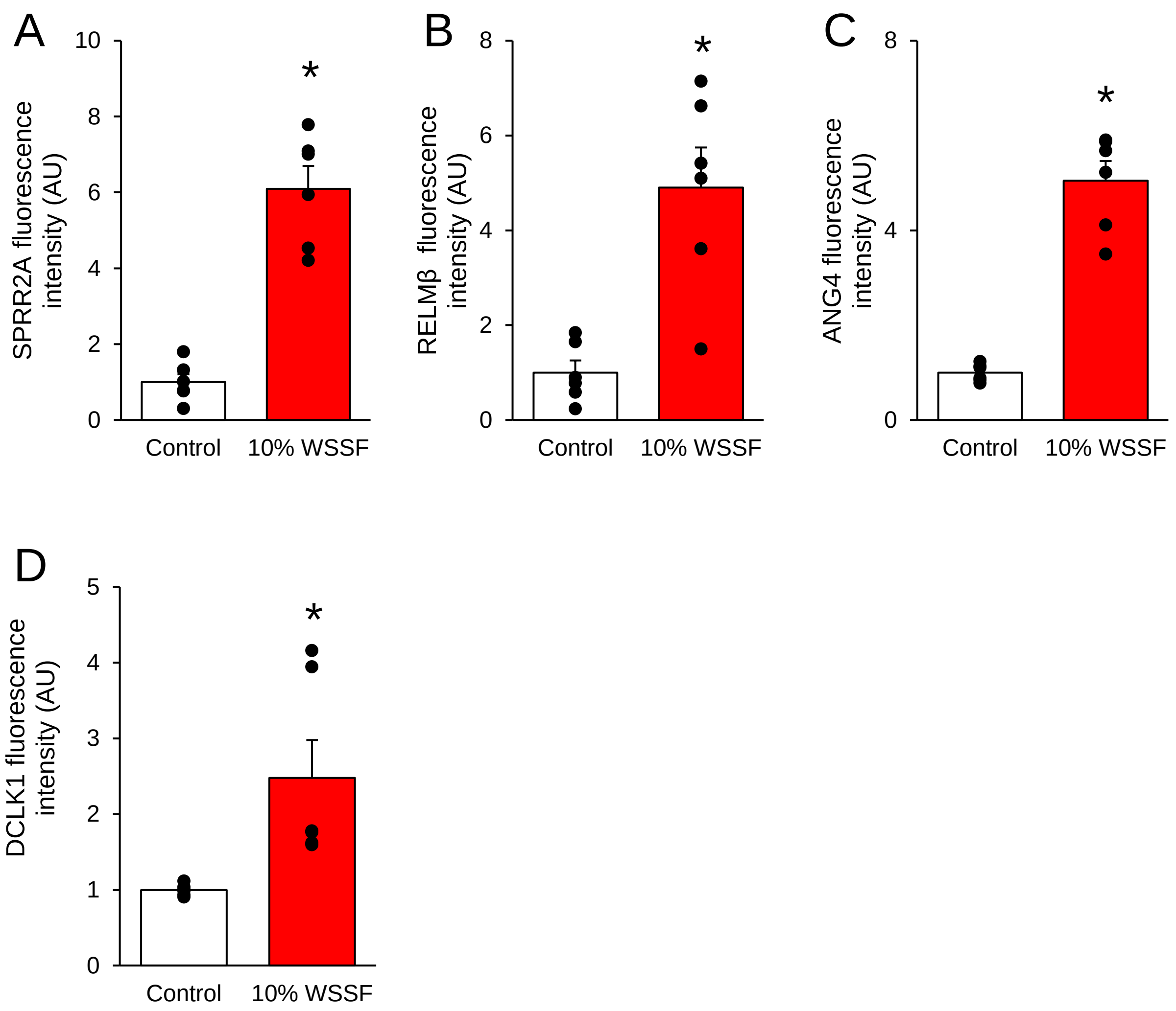

Figure S3. Quantification of SPRR2A, RELMβ, ANG4, and DCLK1 fluorescence intensity in the mouse jejunum from Figures 1K and 3G.

Data are from Experiment 1. Mice were fed control diets or diets supplemented with 10% water-soluble soybean fiber (WSSF) for 5 days. Jejunal cryosections were incubated with primary antibodies, followed by secondary antibodies and 4′,6-diamidino-2-phenylindole (DAPI). Fluorescence intensity was quantified using ImageJ. Data are presented as the mean ± s.e.m. Statistical significance was determined using Mann–Whitney U test or Student's t-test. *$P < 0.05$ vs. control. AU: arbitrary unit. AU, arbitrary units.

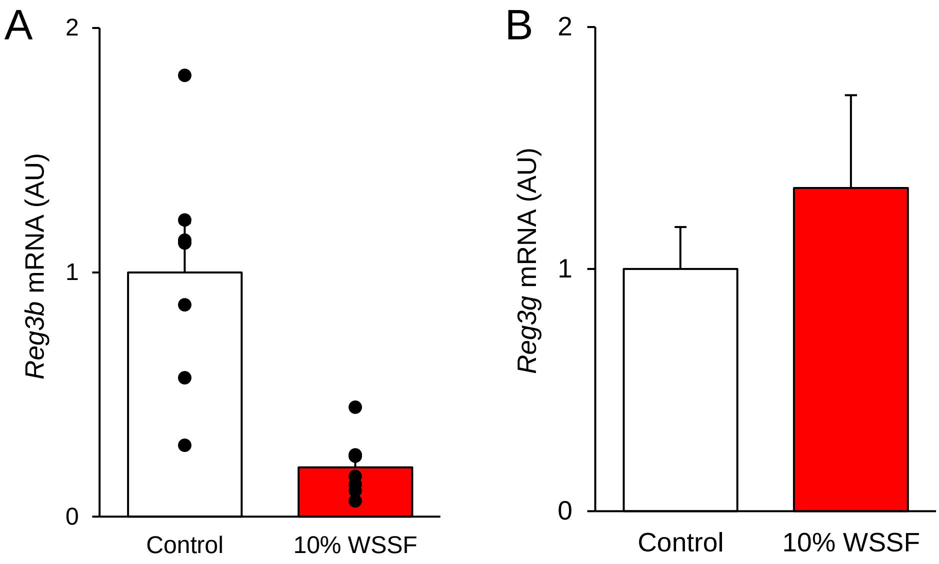

Figure S4. Soybean fiber does not alter Reg3 gene expression in the mouse jejunum.

Data shown are from Experiment 1. Mice were fed control diets or diets supplemented with 10% water-soluble soybean fiber (WSSF) for 5 days, after which jejunal tissues were collected. mRNA expression levels of *Reg3b* and *Reg3g* were quantified by quantitative reverse transcription-PCR (qRT-PCR; A, B). Data are presented as mean ± s.e.m. Statistical significance was assessed using the Tukey–Kramer post hoc test. AU: arbitrary unit.

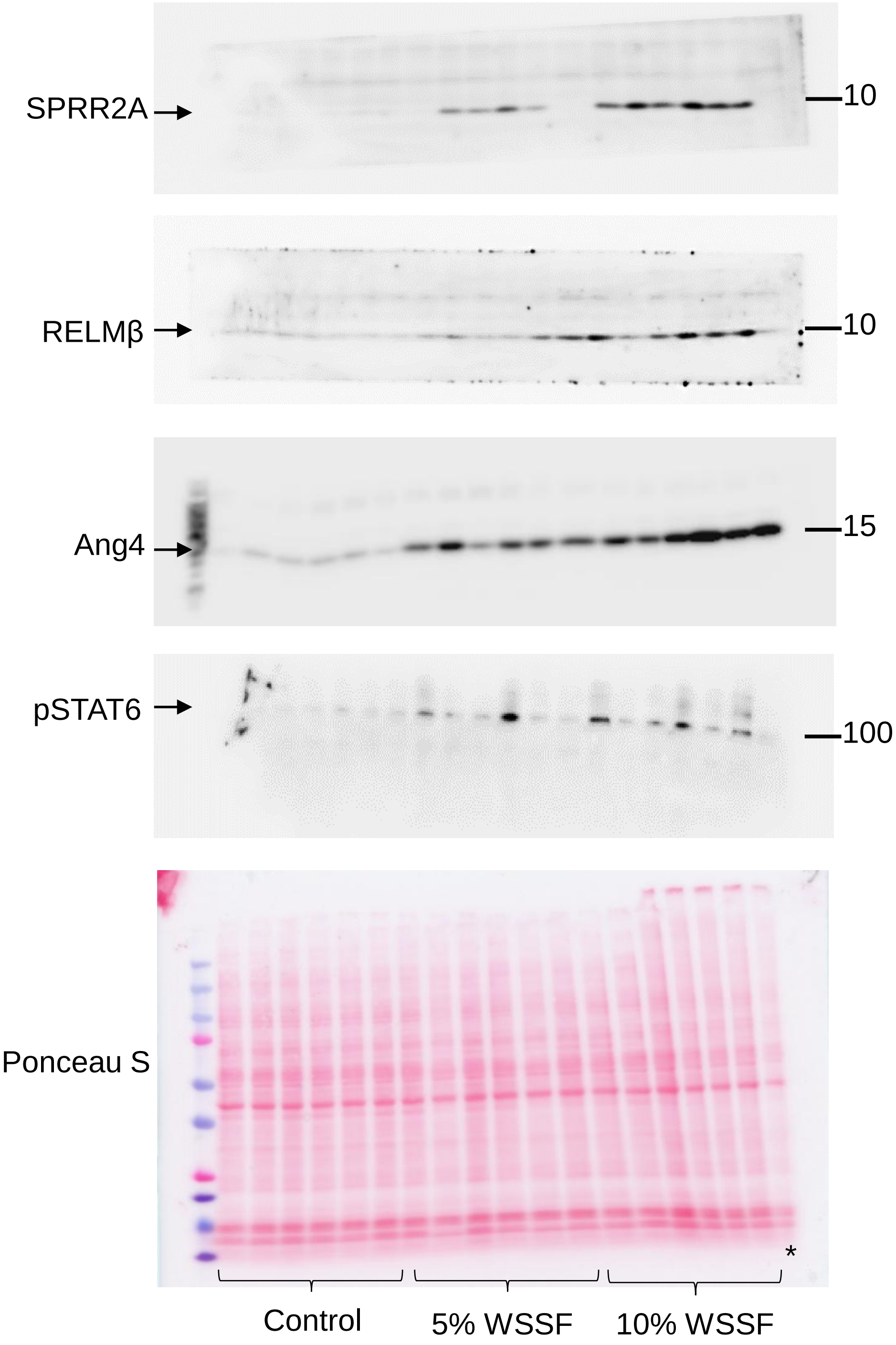

Figure S5. Uncropped original immunoblot images corresponding to the blots shown in Fig. 2 and 3.

Data are from Experiment 1. Mice were fed control diets or diets supplemented with 5% or 10% water-soluble soybean fiber (WSSF) for 5 days. Ileal expression of SPRR2A, RELMβ, ANG4, and pSTAT6 was analyzed by immunoblotting. After transfer to PVDF membranes, total protein loading was visualized by Ponceau S staining and used for normalization. Membranes were cut horizontally according to molecular weight markers and probed separately with primary antibodies. The sample marked with an asterisk is unrelated to the present experiment and was not included in the analysis.

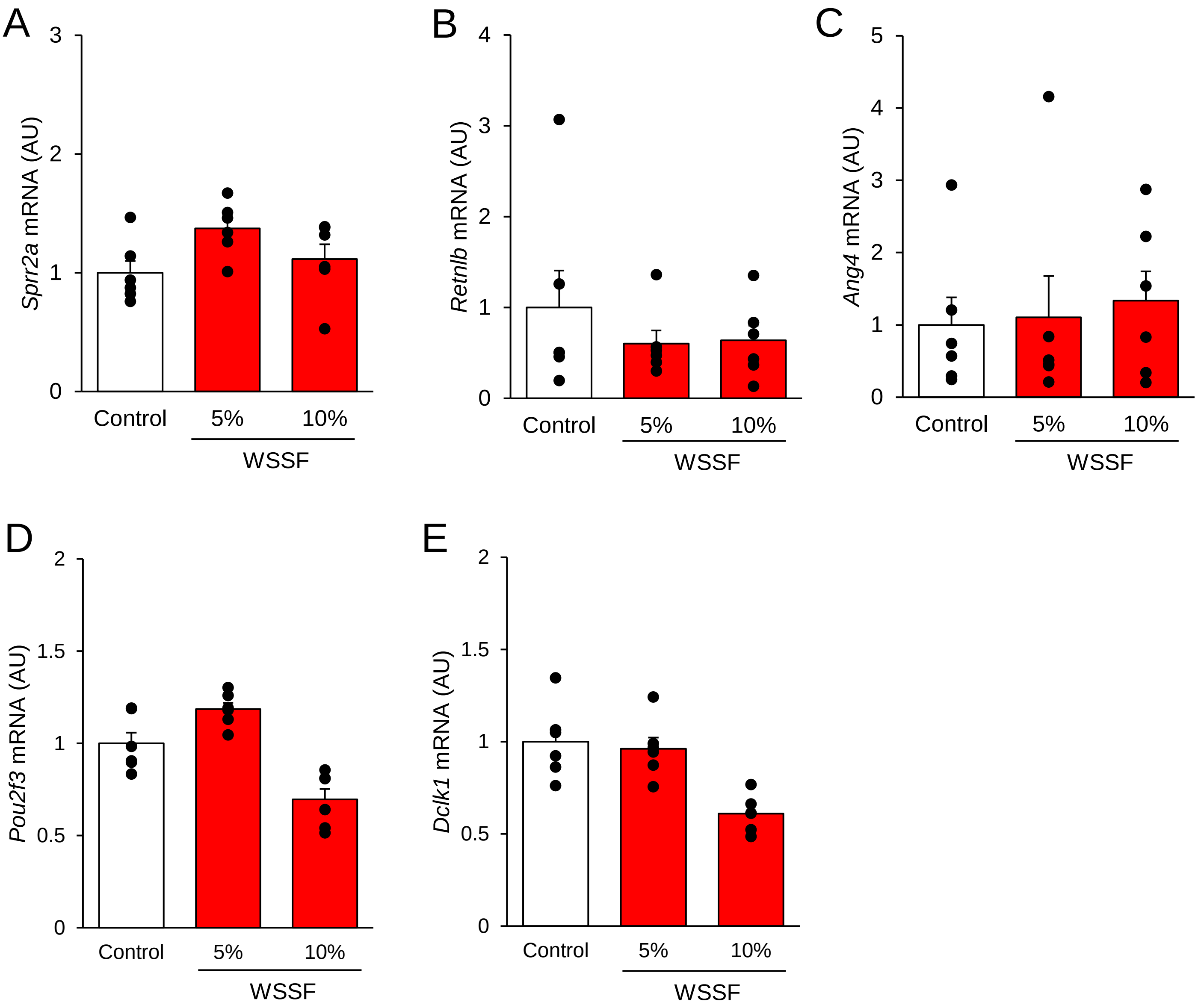

Figure S6. Soybean fiber does not alter gene expression of antimicrobial proteins and tuft cells-related markers in the mouse colon.

Data shown are from Experiment 1. Mice were fed control diets or diets supplemented with 5% or 10% water-soluble soybean fiber (WSSF) for 5 days, after which colonic tissues were collected. mRNA expression levels of *Sprr2a*, *Retnlb*, *Ang4*, *Pou2f3*, and *Dclk1* were quantified by quantitative reverse transcription-PCR (qRT-PCR; A–E). Data are presented as mean ± s.e.m. Statistical significance was determined using the Tukey–Kramer or Steel–Dwass test. AU: arbitrary unit.

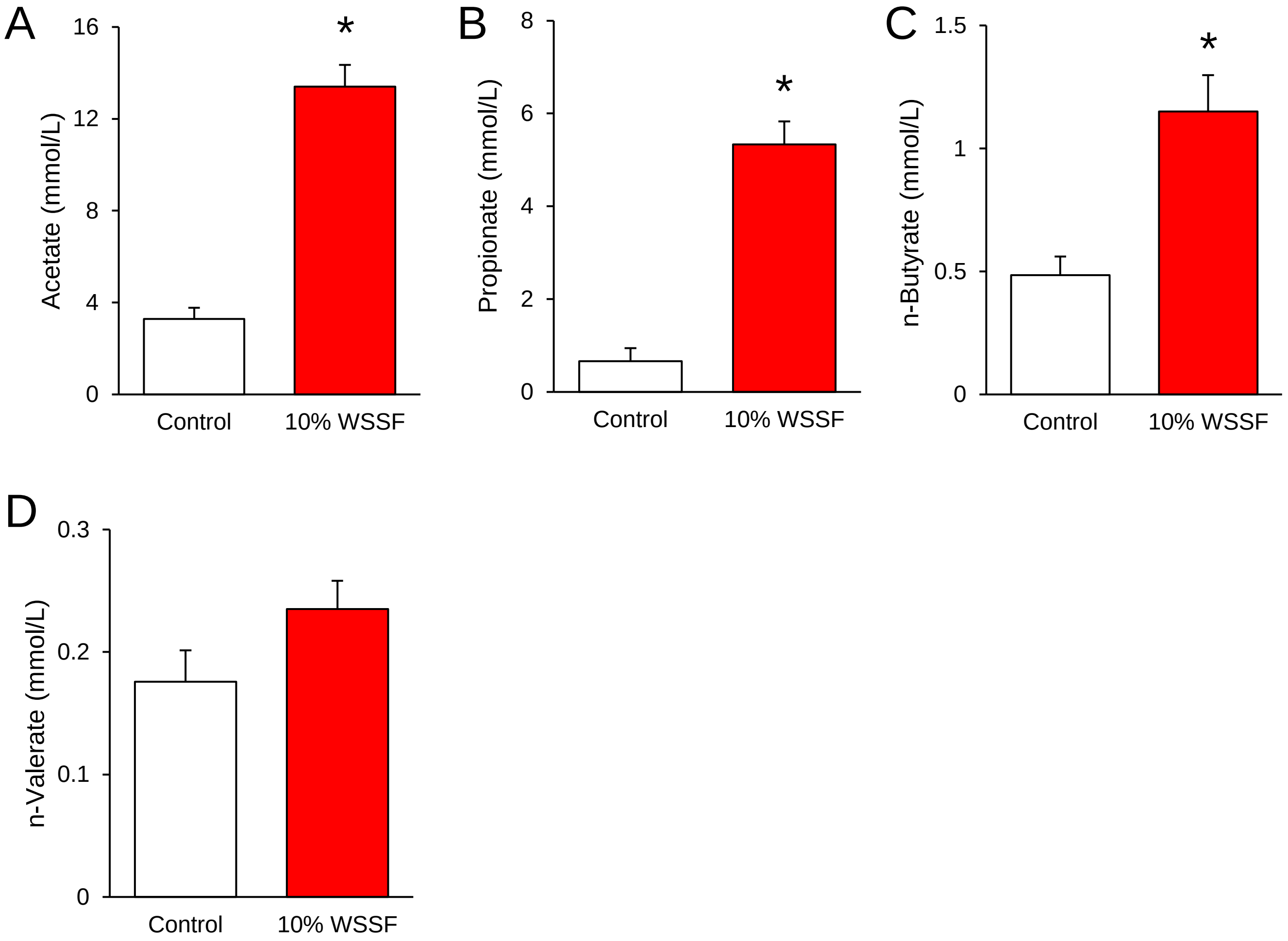

Figure S7. Soybean fiber increases short-chain fatty acids in the mouse feces.
Data shown are from Experiment 1. Mice were fed control diets or diets supplemented with 10% water-soluble soybean fiber (WSSF) for 5 days, after which fecal samples were collected. The levels of acetate, propionate, n-butyrate and n-valeratee were measured (A-D). Data are presented as mean ± s.e.m. Statistical significance was determined using Mann–Whitney U test or Student's t-test. *$P < 0.05$ vs. control.

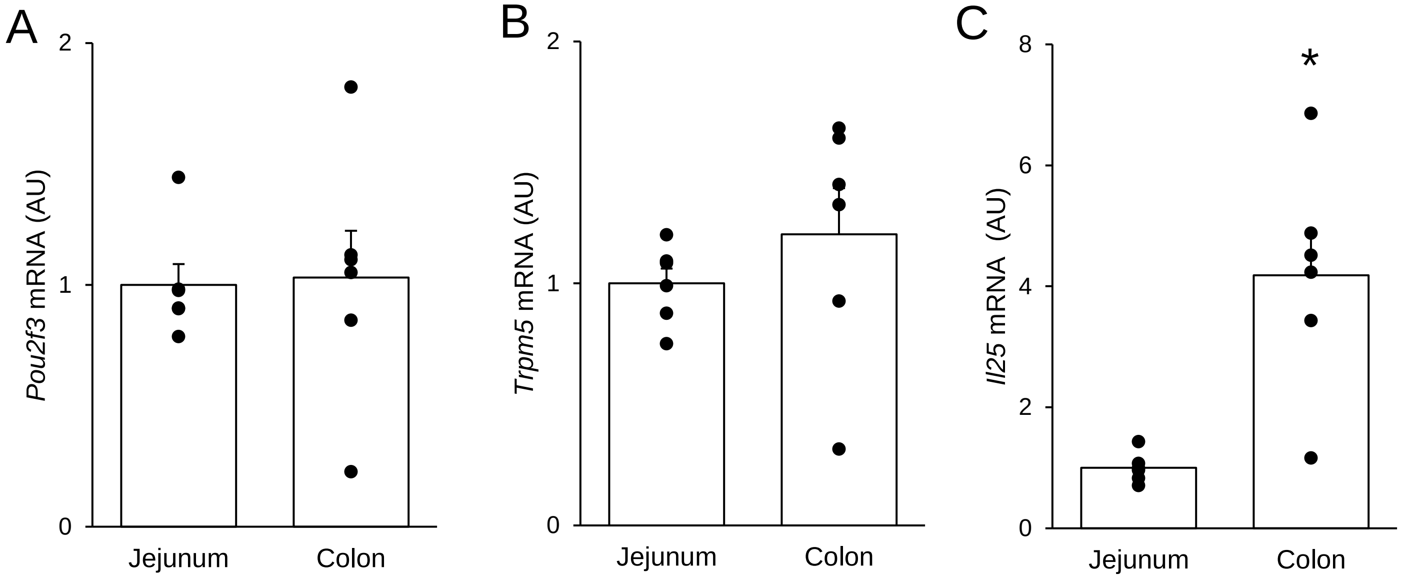

Figure S8. Expression of tuft cell-related genes in the mouse jejunum and colon.
Data shown are from Experiment 1. Mice were fed control diet for 5 days, after which jejunal and colonic tissues were collected. mRNA expression of *Pou2f3*, *Trpm5*, and *Il25* were quantified by quantitative reverse transcription-PCR (qRT-PCR; A–C). Data are presented as mean ± s.e.m. Statistical significance was determined using Mann–Whitney U test or Student's t-test. *$P < 0.05$ vs. control. AU: arbitrary unit.

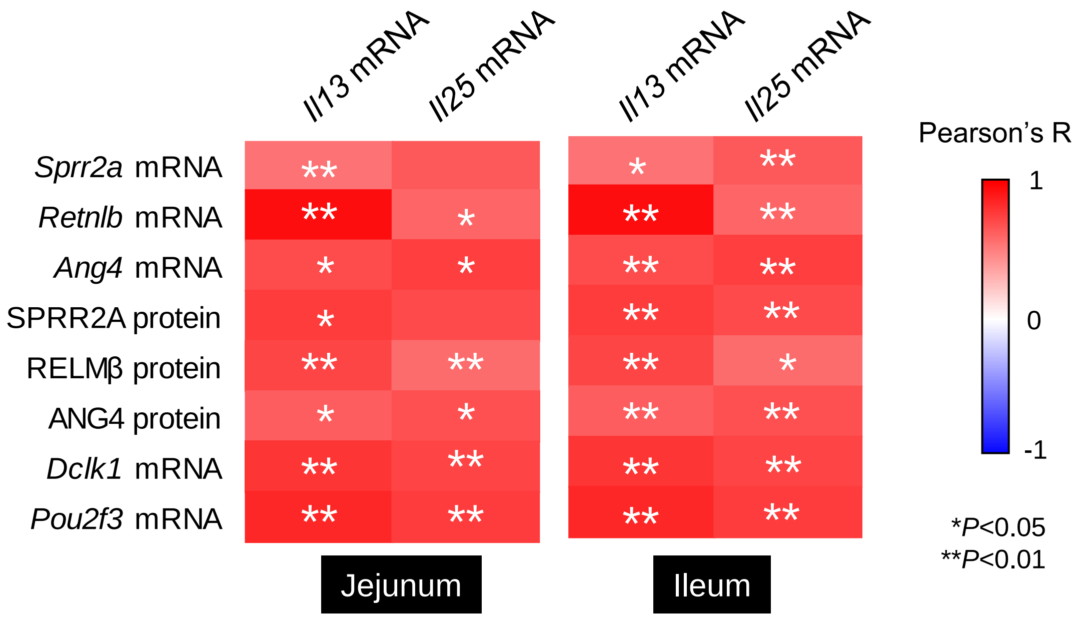

Figure S9. Pearson correlation analysis of mRNA expression of antimicrobial proteins, tuft cell markers, and type 2 cytokines in the mouse jejunum and ileum.

Data shown are from Experiment 1. Mice were fed control diets or diets supplemented with 5% or 10% water-soluble soybean fiber (WSSF) for 5 days, after which jejunal and ileal tissues were subjected to quantitative reverse transcription-PCR (qRT-PCR) analysis. Correlation coefficients are shown by the color scale. Asterisks denote significant correlations between variables (**P* < 0.05, ***P* < 0.01).

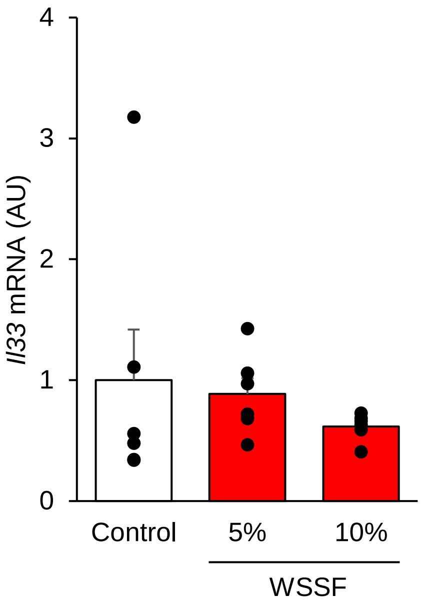

Figure S10. Soybean fiber does not alter *Il33* gene expression in the mouse jejunum.

Data shown are from Experiment 1. Mice were fed control diets or diets supplemented with 5% or 10% water-soluble soybean fiber (WSSF) for 5 days, after which jejunal tissues were collected. Il33 mRNA expression was quantified by quantitative real-time PCR (qRT-PCR). Data are presented as mean ± s.e.m. Statistical significance was assessed using the Tukey–Kramer post hoc test. AU: arbitrary unit.

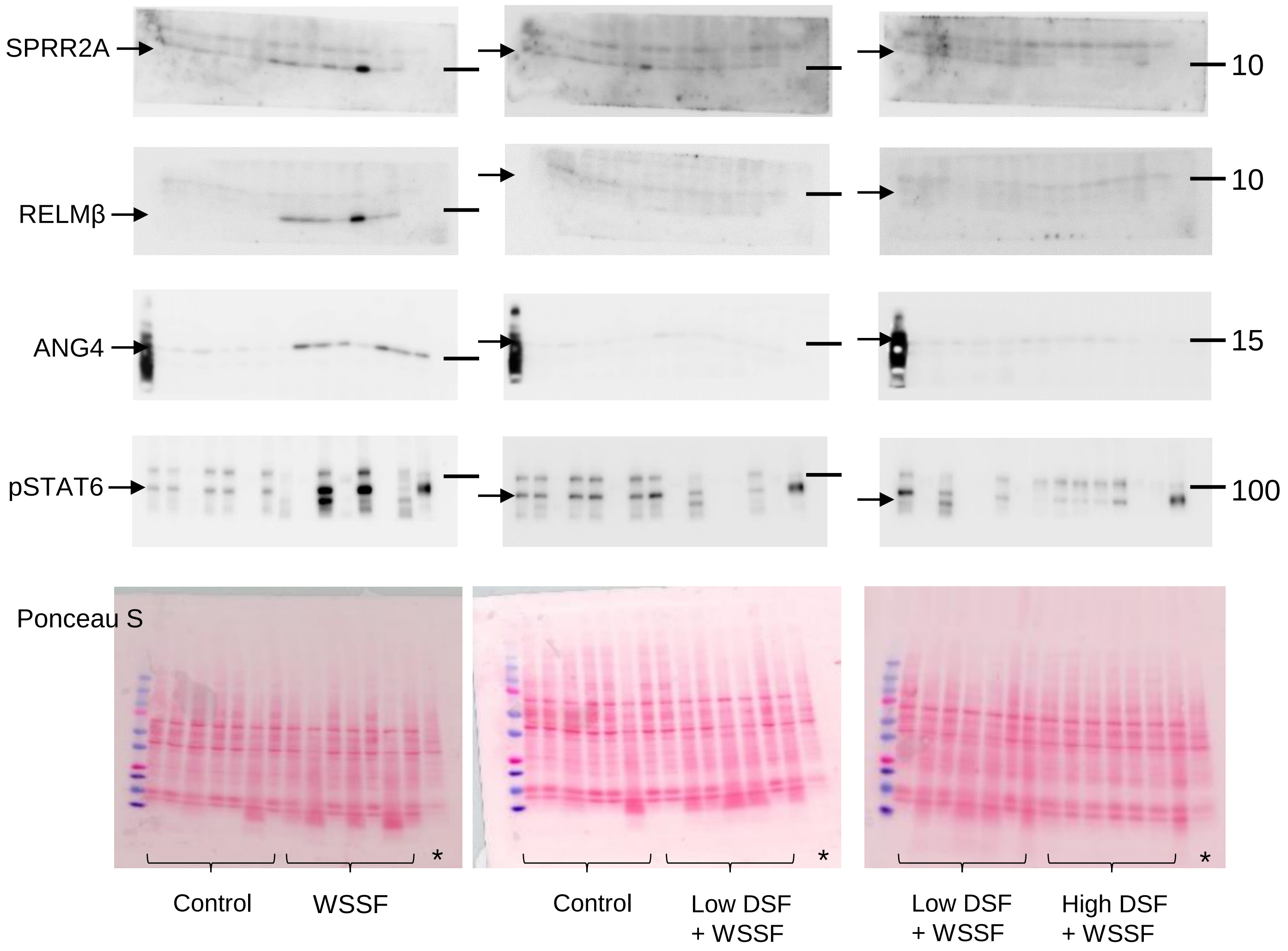

Figure S11. Uncropped original immunoblot images corresponding to the blots shown in Fig. 4 and 5.
Data are from Experiment 2. Mice were fed control diets or diets supplemented with 10% water-soluble soybean fiber (WSSF) for 5 days. Mice receiving the WSSF-containing diet were treated with the ILC2 inhibitor disulfiram (DSF) at two doses (low and high). Jejunal expression of SPRR2A, RELMβ, ANG4, and pSTAT6 was analyzed by immunoblotting. After transfer to PVDF membranes, total protein loading was visualized by Ponceau S staining and used for normalization. Membranes were cut horizontally according to molecular weight markers and probed separately with primary antibodies. The sample marked with an asterisk is unrelated to the present experiment and was not included in the analysis.

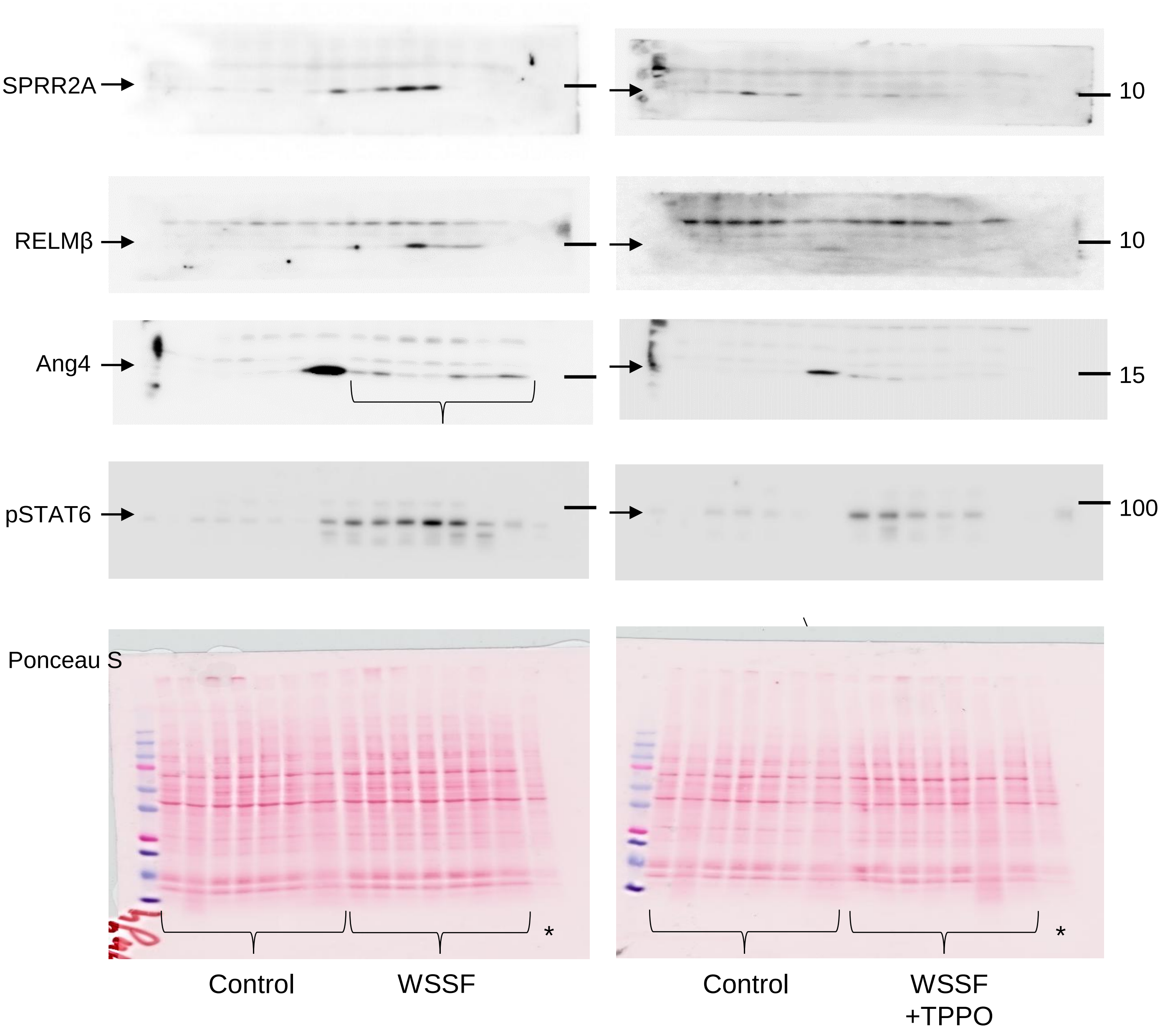

Figure S12. Uncropped original immunoblot images corresponding to the blots shown in Fig. 6 and 7.

Data are from Experiment 3. Mice were fed control diets or diets supplemented with 10% water-soluble soybean fiber (WSSF) for 5 days. Mice receiving the WSSF-containing diet were treated with the TRPM5 inhibitor triphenylphosphine oxide (TPPO). Jejunal expression of SPRR2A, RELMβ, ANG4, and pSTAT6 was analyzed by immunoblotting. After transfer to PVDF membranes, total protein loading was visualized by Ponceau S staining and used for normalization. Membranes were cut horizontally according to molecular weight markers and probed separately with primary antibodies. The sample marked with an asterisk is unrelated to the present experiment and was not included in the analysis.

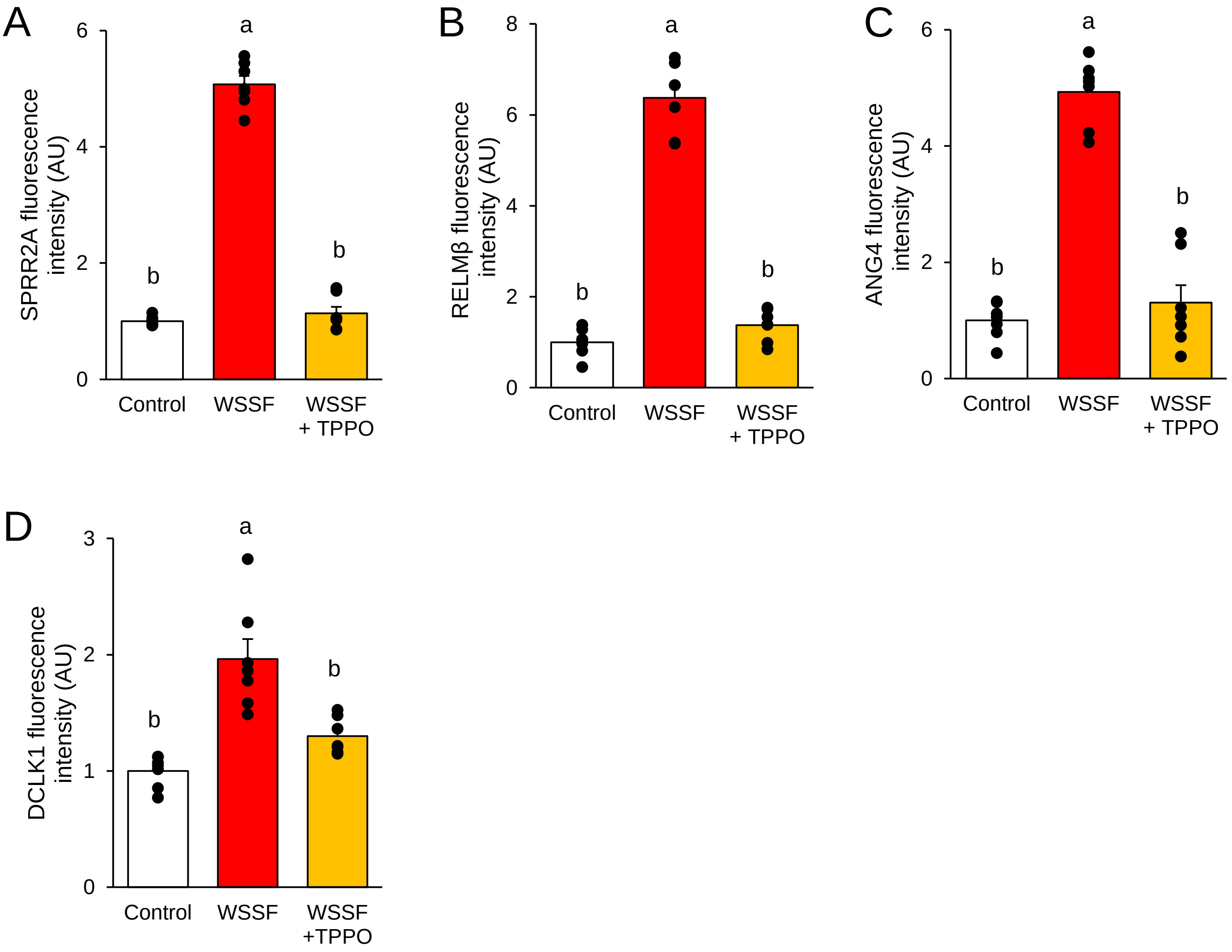

Figure S13. Quantification of SPRR2A, RELMβ, ANG4, and DCLK1 fluorescence intensity in the mouse jejunum from Figures 6H and 7F.
Data are from Experiment 3. Mice were fed control diets or diets supplemented with 10% water-soluble soybean fiber (WSSF) for 5 days. Mice receiving the WSSF-containing diet were treated with the TRPM5 inhibitor triphenylphosphine oxide (TPPO). Jejunal cryosections were incubated with primary antibodies, followed by secondary antibodies and 4′,6-diamidino-2-phenylindole (DAPI). Fluorescence intensity was quantified using ImageJ. Data are presented as the mean ± s.e.m. Statistical significance was assessed using the Tukey–Kramer post hoc test or the Steel–Dwass test. Groups not sharing a common letter are significantly different ($p < 0.05$). AU, arbitrary units.

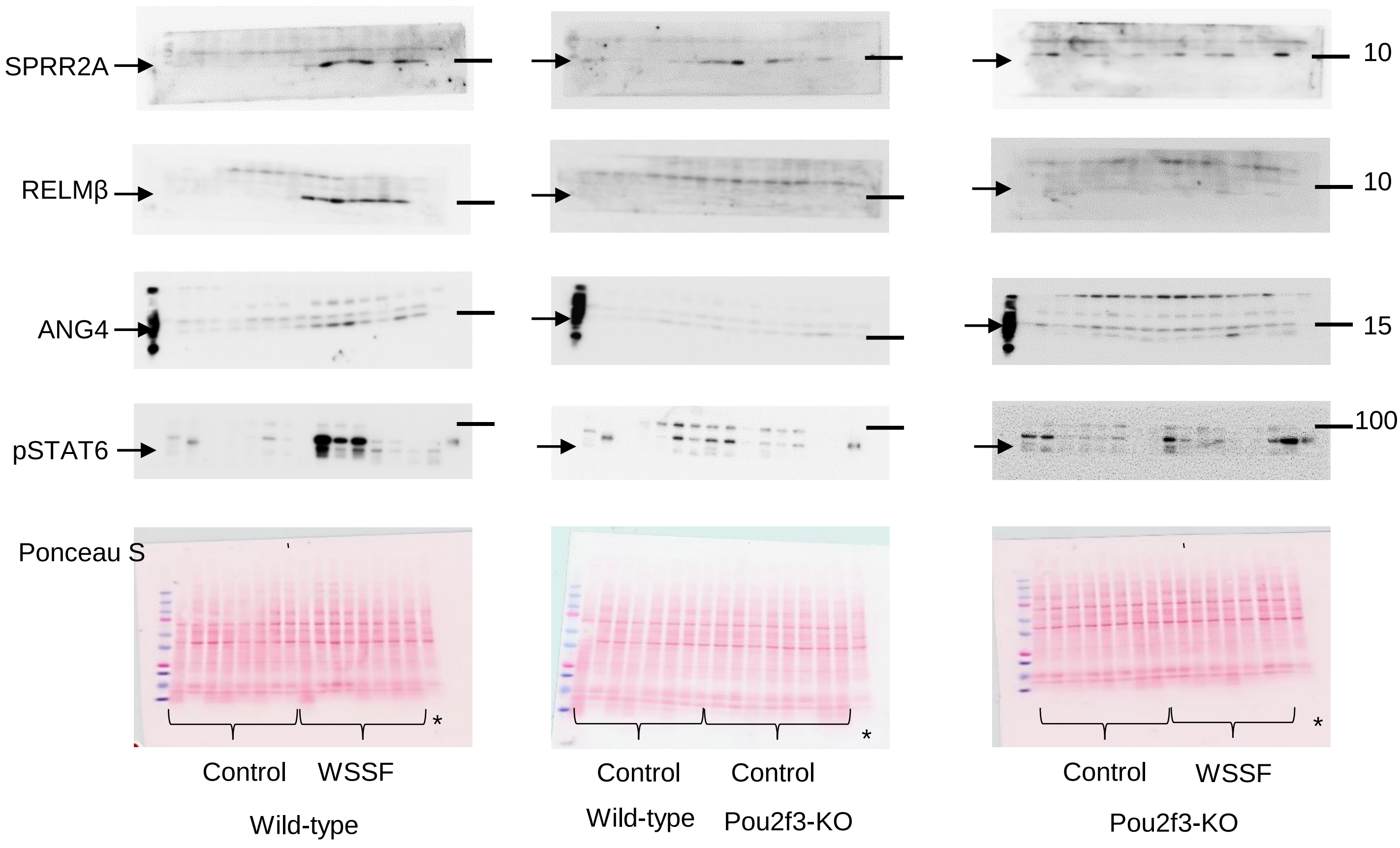

Figure S14. Uncropped original immunoblot images corresponding to the blots shown in Fig. 8 and 9.

Data are from Experiment 4. *Pou2f3*-KO mice and wild-type littermates were fed control diets or diets supplemented with 10% water-soluble soybean fiber (WSSF) for 5 days. Jejunal expression of SPRR2A, RELMβ, ANG4, and pSTAT6 was analyzed by immunoblotting. After transfer to PVDF membranes, total protein loading was visualized by Ponceau S staining and used for normalization. Membranes were cut horizontally according to molecular weight markers and probed separately with primary antibodies. The sample marked with an asterisk is unrelated to the present experiment and was not included in the analysis. KO: knockout.

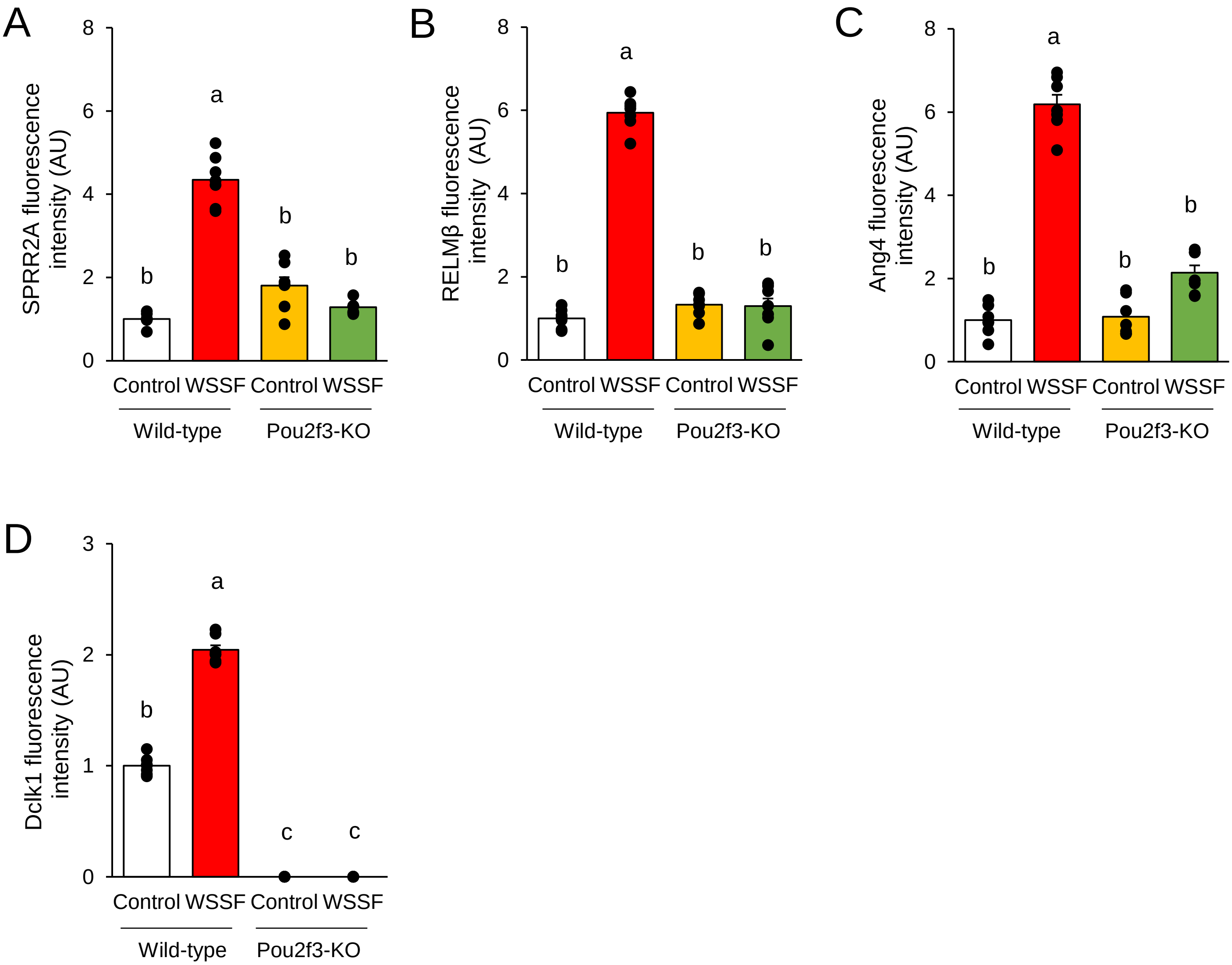

Figure S15. Quantification of SPRR2A, RELMβ, ANG4, and DCLK1 fluorescence intensity in the mouse jejunum from Figures 8H and 9F.

Data are from Experiment 4. *Pou2f3*-KO mice and wild-type littermates were fed control diets or diets supplemented with 10% water-soluble soybean fiber (WSSF) for 5 days. Jejunal cryosections were incubated with primary antibodies, followed by secondary antibodies and 4′,6-diamidino-2-phenylindole (DAPI). Fluorescence intensity was quantified using ImageJ. Data are presented as the mean ± s.e.m. Statistical significance was assessed using the Tukey–Kramer post hoc test or the Steel–Dwass test. Groups not sharing a common letter are significantly different ($p < 0.05$). AU, arbitrary units; KO: knockout.

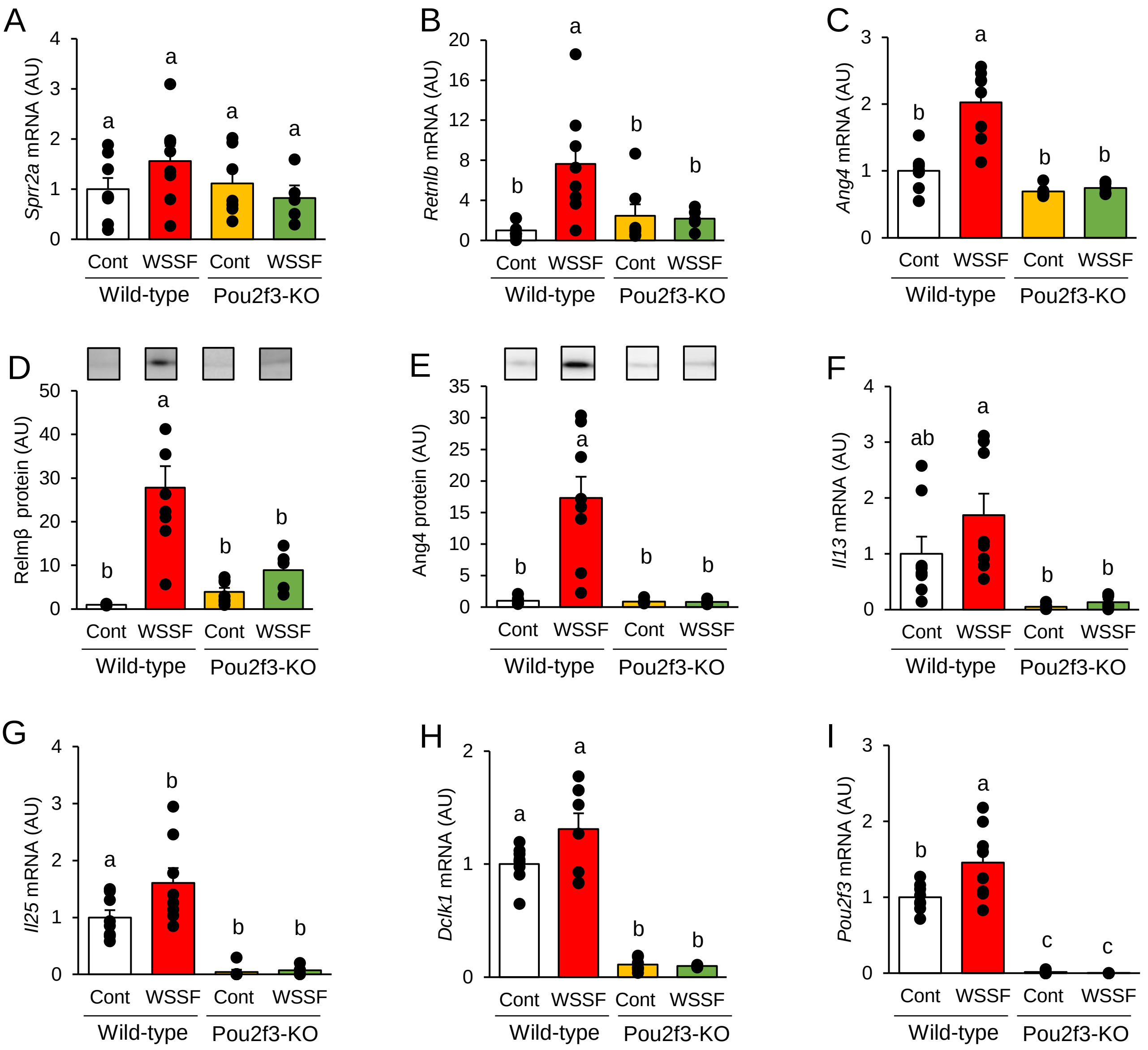

Figure S16. Tuft cells are required for soybean fiber–mediated antimicrobial protein expression and upregulation of tuft cell markers and type 2 immunity–related molecules in the mouse ileum.

Data shown are from Experiment 4. *Pou2f3*-KO mice and wild-type littermates were fed control diets or diets supplemented with 10% water-soluble soybean fiber (WSSF) for 5 days, after which ileal tissues were collected. mRNA expression levels of *Sprr2a* (A), *Retnlb* (B), *Ang4* (C), *Il33* (F), *Il25* (G), *Dclk1* (H), and *Pou2f3* (I) were quantified by quantitative reverse transcription-PCR (qRT-PCR). Protein levels of RELMβ (D) and ANG4 (E) were evaluated by immunoblotting. Data are presented as mean ± s.e.m. Statistical significance was assessed using the Tukey–Kramer post hoc test or the Steel–Dwass test. Groups not sharing a common letter are significantly different ($p < 0.05$). AU: arbitrary unit; KO: knockout.

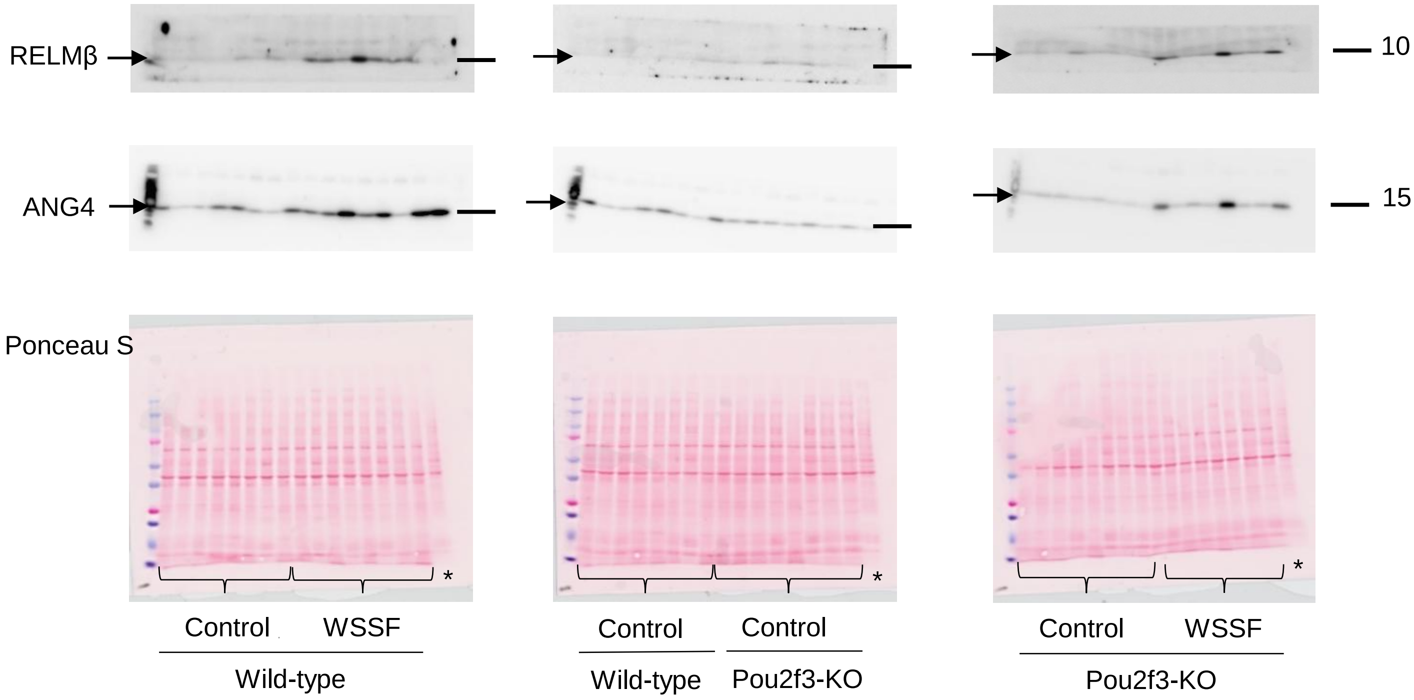

Figure S17. Uncropped original immunoblot images corresponding to the blots shown in Fig. S16. Data are from Experiment 4. *Pou2f3*-KO mice and wild-type littermates were fed control diets or diets supplemented with 10% water-soluble soybean fiber (WSSF) for 5 days. Jejunal expression of SPRR2A, RELMβ, and ANG4 was analyzed by immunoblotting. After transfer to PVDF membranes, total protein loading was visualized by Ponceau S staining and used for normalization. Membranes were cut horizontally according to molecular weight markers and probed separately with primary antibodies. The sample marked with an asterisk is unrelated to the present experiment and was not included in the analysis. KO: knockout

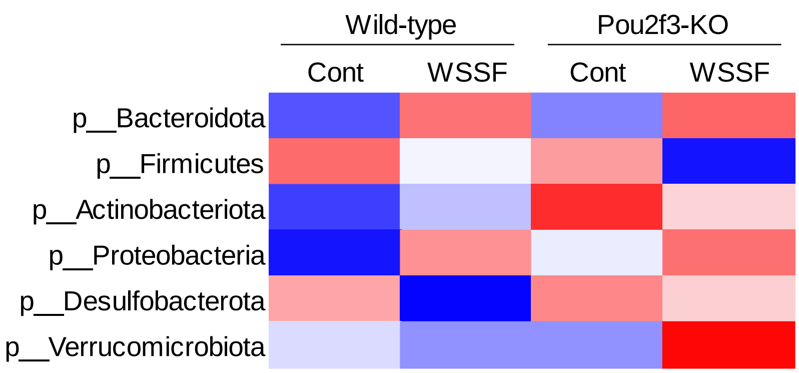

	2-way ANOVA		
	Diet	Genotype	D x G
p__Bacteroidota	**		
p__Firmicutes	**	*	
p__Actinobacteriota		*	
p__Proteobacteria	**	*	
p__Desulfobacterota			
p__Verrucomicrobiota			

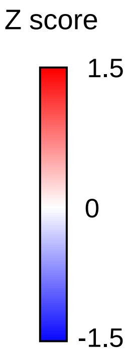

*P<0.05
**P<0.01

Figure S18. Two-way ANOVA of phylum-level abundances in the cecal microbiota of mice fed soybean fiber.

Data shown are from Experiment 4. *Pou2f3*-KO mice and wild-type littermates were fed control diets or diets supplemented with 10% water-soluble soybean fiber (WSSF) for 5 days, after which cecal contents were collected. Cecal microbiota composition was analyzed by 16S rRNA gene sequencing, and sequence data were processed using QIIME 2. Two-way ANOVA (analysis of variance) was performed at the phylum level. A heatmap based on Z-scores of relative abundances at the phylum level is shown. Relative abundances were log-transformed after adding 1×10^{-6}, and Z-scores were calculated. Asterisks indicate statistically significant effects of soybean fiber (D: Diet), genotype (G), or their interaction (*P < 0.05, **P < 0.01).

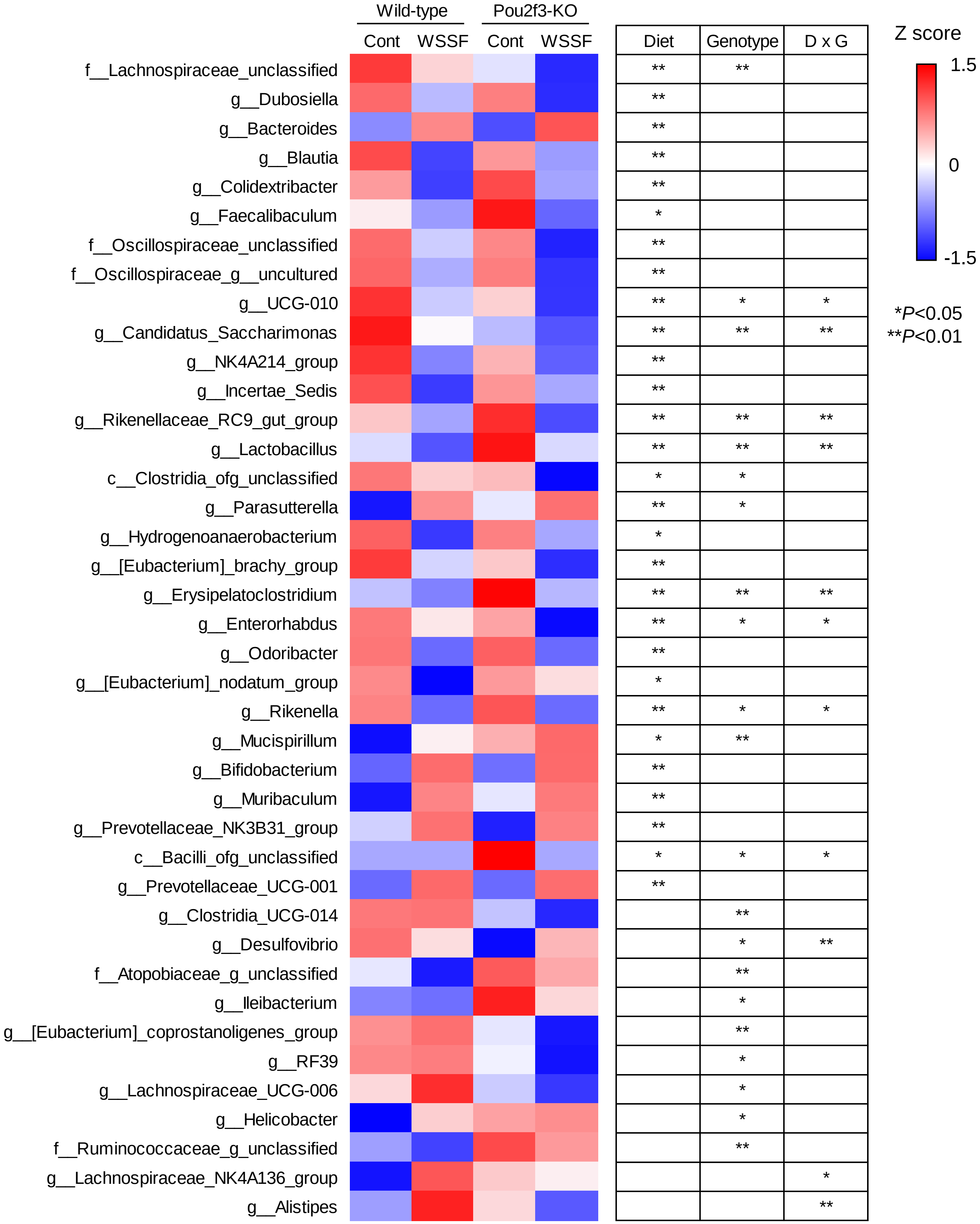

	Diet	Genotype	D x G
f__Lachnospiraceae_unclassified	**	**	
g__Dubosiella	**		
g__Bacteroides	**		
g__Blautia	**		
g__Colidextribacter	**		
g__Faecalibaculum	*		
f__Oscillospiraceae_unclassified	**		
f__Oscillospiraceae_g__uncultured	**		
g__UCG-010	**	*	*
g__Candidatus_Saccharimonas	**	**	**
g__NK4A214_group	**		
g__Incertae_Sedis	**		
g__Rikenellaceae_RC9_gut_group	**	**	**
g__Lactobacillus	**	**	**
c__Clostridia_ofg_unclassified	*	*	
g__Parasutterella	**	*	
g__Hydrogenoanaerobacterium	*		
g__[Eubacterium]_brachy_group	**		
g__Erysipelatoclostridium	**	**	**
g__Enterorhabdus	**	*	*
g__Odoribacter	**		
g__[Eubacterium]_nodatum_group	*		
g__Rikenella	**	*	*
g__Mucispirillum	*	**	
g__Bifidobacterium	**		
g__Muribaculum	**		
g__Prevotellaceae_NK3B31_group	**		
c__Bacilli_ofg_unclassified	*	*	*
g__Prevotellaceae_UCG-001	**		
g__Clostridia_UCG-014		**	
g__Desulfovibrio		*	**
f__Atopobiaceae_g_unclassified		**	
g__Ileibacterium		*	
g__[Eubacterium]_coprostanoligenes_group		**	
g__RF39		*	
g__Lachnospiraceae_UCG-006		*	
g__Helicobacter		*	
f__Ruminococcaceae_g_unclassified		**	
g__Lachnospiraceae_NK4A136_group			*
g__Alistipes			**

Figure S19. Two-way ANOVA of genus-level abundances in the cecal microbiota of mice fed soybean fiber. Data shown are from Experiment 4. *Pou2f3*-KO mice and wild-type littermates were fed control diets or diets supplemented with 10% water-soluble soybean fiber (WSSF) for 5 days, after which cecal contents were collected. Cecal microbiota composition was analyzed by 16S rRNA gene sequencing, and sequence data were processed using QIIME 2. Two-way ANOVA was performed at the genus level. A heatmap based on Z-scores of relative abundances at the genus level is shown. Relative abundances were log-transformed after adding 1×10^{-6}, and Z-scores were calculated. Asterisks indicate statistically significant effects of soybean fiber (D: Diet), genotype (G), or their interaction (*$P < 0.05$, **$P < 0.01$). Genera without significant effects are not shown. KO: knockout.

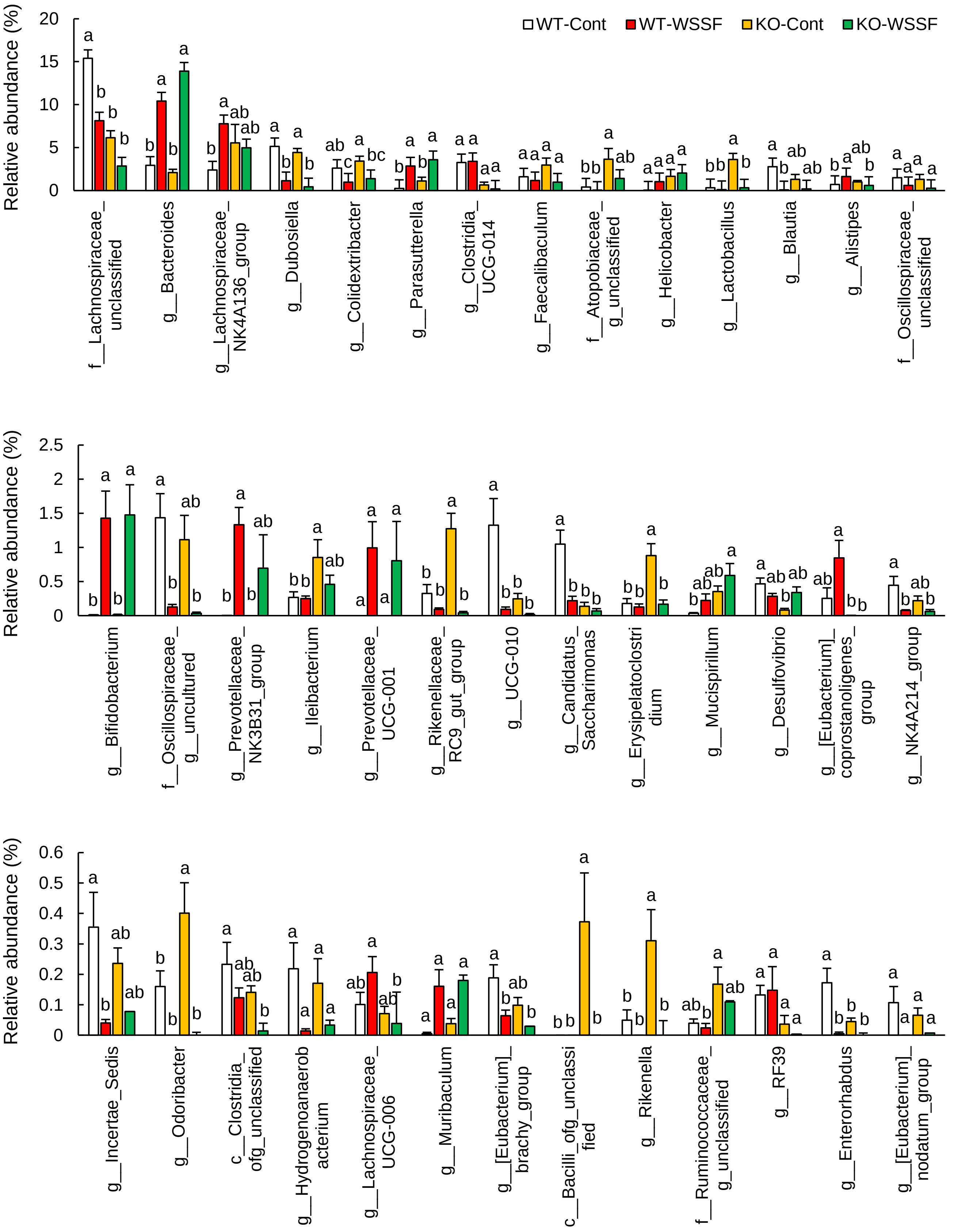

Figure S20. Microbiota composition at the genus level in cecal contents of mice fed soybean fiber. Data shown are from Experiment 4. *Pou2f3*-KO mice and wild-type littermates were fed control diets or diets supplemented with 10% water-soluble soybean fiber (WSSF) for 5 days, after which cecal contents were collected. Cecal microbiota composition was analyzed by 16S rRNA gene sequencing, and sequence data were processed using QIIME 2. Mean relative abundances of cecal microbiota at the genus level are shown. Data are presented as mean ± s.e.m. (n = 8). Statistical significance was assessed using the Tukey–Kramer post hoc test or the Steel–Dwass test. Groups not sharing a common letter are significantly different ($p < 0.05$). KO: knockout.

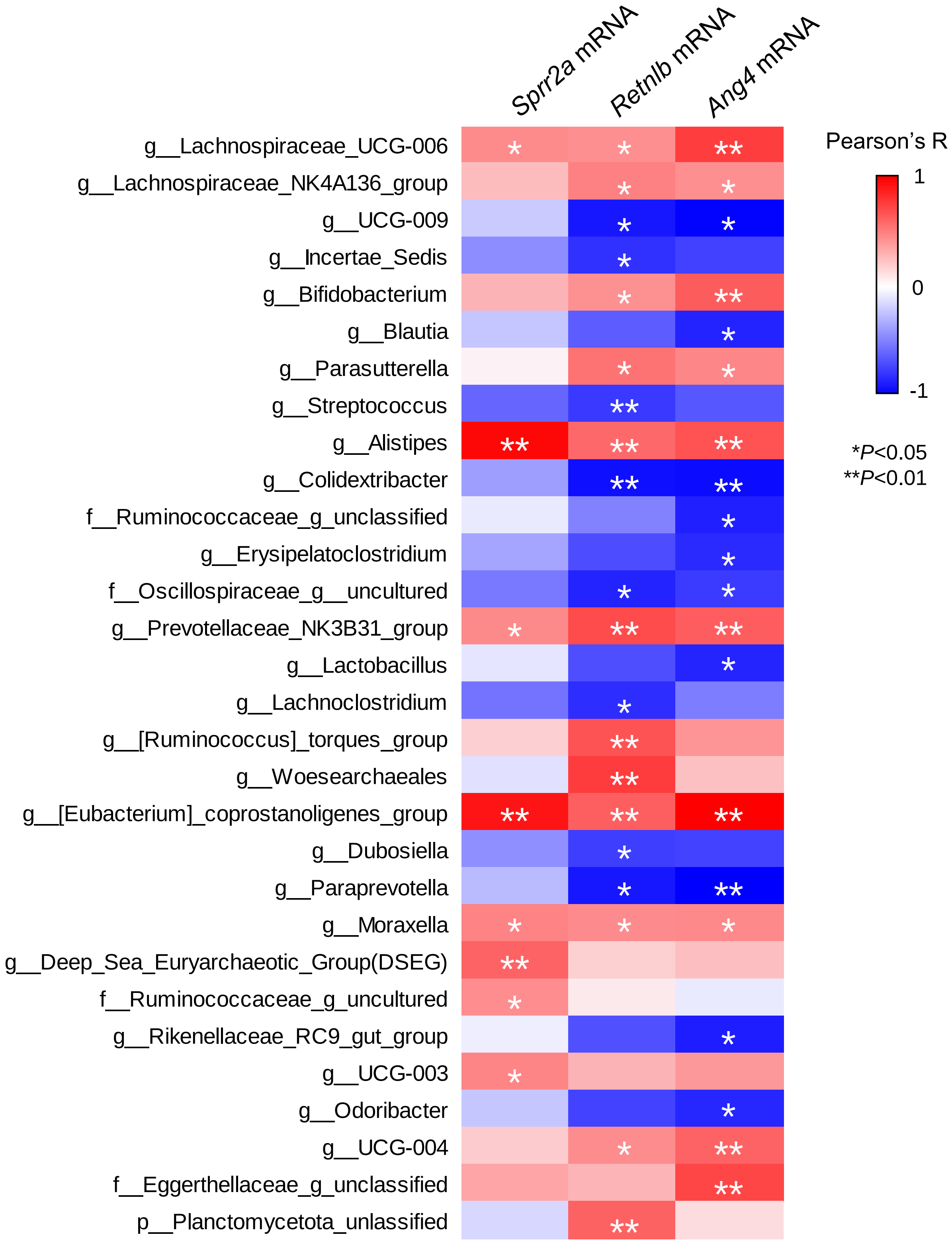

Figure S21. Pearson correlation analysis between jejunal antimicrobial protein gene expression and cecal microbiota composition.

Data shown are from Experiment 4. *Pou2f3*-KO mice and wild-type littermates were fed control diets or diets supplemented with 10% water-soluble soybean fiber (WSSF) for 5 days. Correlation coefficients are indicated by the color scale. Asterisks denote significant correlations between variables (**P* < 0.05, ***P* < 0.01). KO: knockout.
